# Impact of menopausal status on human papillomavirus detection and cervical neoplasia in middle-aged Senegalese women

**DOI:** 10.1371/journal.pone.0358080

**Published:** 2026-09-11

**Authors:** Emily R. Spencer, Rachel L. Winer, Selly Ba, Marie Pierre Sy, John Lin, Qinghua Feng, Geoffrey S. Gottlieb, Papa Salif Sow, Nancy B. Kiviat, Stephen E. Hawes

**Affiliations:** 1 Department of Epidemiology, University of Washington, Seattle, Washington, United States of America; 2 Service des Maladies Infectieuses Centre Hospitalier National Universitaire (CHNU) de Fann, Dakar, Sénégal; 3 Department of Pathology, School of Medicine, University of Washington, Seattle, Washington, United States of America; 4 Division of Allergy and Infectious Diseases, School of Medicine, University of Washington, Seattle, Washington, United States of America; 5 Department of Global Health, University of Washington, Seattle, Washington, United States of America; UFRN: Universidade Federal do Rio Grande do Norte, BRAZIL

## Abstract

Hormonal and immunologic changes related to menopause may play a role in human papillomavirus (HPV) and cervical neoplasia detection, yet this relationship has not been examined in West African populations, where cervical cancer remains a leading cause of cancer-related death. The purpose of this study was to assess whether menopausal status is associated with HPV and cervical neoplasia detection in Senegalese women aged 40–60. We conducted a secondary analysis of three prospective cervical cancer studies in Dakar, Senegal (1998–2011). Participants with known menopausal status and a satisfactory HPV test (N = 3,118) and/or Pap smear (N = 2,907) were included, excluding those with cervical cancer or pregnancy; participants without known HIV status were additionally excluded from the cervical neoplasia analysis. Multivariable logistic regression and generalized estimating equations were used to estimate adjusted odds ratios and 95% confidence intervals, adjusting for age, gravidity, and HIV status. Compared with premenopausal women, postmenopausal women had significantly higher odds of HPV detection (OR = 1.48; 95% CI: 1.18, 1.85), including both high-risk (OR = 1.55; 95% CI: 1.25, 1.93) and low-risk types (OR = 1.64; 95% CI: 1.30, 2.08). Odds of cervical neoplasia were elevated, but non-significant, among postmenopausal women (OR = 1.36; 95% CI: 0.97, 1.90), with increased odds for both high-grade (OR = 1.43; 95% CI: 0.90, 2.28) and low-grade lesions (OR = 1.30; 95% CI: 0.84, 2.02). However, in a sensitivity analysis restricted to participants with known HIV status, these cervical neoplasia odds were attenuated, suggesting caution in interpreting the magnitude of these findings. These findings suggest menopause may contribute to HPV persistence or reactivation, and development of cervical neoplasia. Our results highlight the importance of continued surveillance for HPV and cervical neoplasia among older women, particularly in low-resource settings with disproportionately high cervical cancer burden.

## Introduction

Cervical cancer is the fourth most common malignancy among women globally, disproportionately affecting low- and middle-income countries (LMICs), which account for 85% of cervical cancer deaths [1,2]. Over the next two decades, an estimated 11 million women in LMICs will be diagnosed with cervical cancer, many of whom will be of menopausal age [2,3]. In Senegal, cervical cancer is the most prevalent cancer among women, with an incidence rate of 34.7 cases per 100,000 woman years, 261% higher than the global average [4–6], and is the leading cause of cancer-related mortality [7]. This figure likely underestimates the true burden, as many cases remain undiagnosed due to gaps in screening and healthcare infrastructure.

Persistent infection with high-risk oncogenic types of human papillomavirus (HPV) is responsible for roughly 99.7% of cervical cancer cases [1]. HPV infection can cause precancerous lesions, which if left unidentified and/or untreated, may develop into invasive cancers [1]. Although vaccination programs have successfully reduced HPV infection [8,9] and cervical cancer [10] in some populations, their reach in West Africa remains limited. As of 2021, only nine countries in the region have initiated pilot programs, focusing on the vaccination of girls aged 9–14, and only Senegal and Gambia have implemented national vaccination programs. Despite these programs, only about 20% of Senegalese girls have been vaccinated, and just 10% of women aged 30–69 have ever been screened for cervical cancer [6,11,12]. This underscores the ongoing need for screening to reduce cervical cancer risk, even with vaccination efforts in place.

While public health efforts have largely focused on vaccinating adolescent girls [13,14], older women remain at risk. HPV prevalence in women often displays a bimodal distribution, with a primary peak in early adulthood and a second, smaller peak in the fifth or sixth decade of life [15–18]. One proposed explanation for this secondary rise is the hormonal and immunologic changes associated with menopause [19,20]. Postmenopausal women experience reduced immune function and increased vaginal dysbiosis, which may impair their ability to clear both new and recurring HPV infections [21–24]. In addition, age-related changes to the cervix may affect lesion development and detection [19,20]. Despite these potential mechanisms, few studies have directly examined the role of menopause in HPV infection or cervical neoplasia development.

Evidence from populations outside of Africa is inconsistent. Some studies report no association between menopausal status and HPV prevalence [22,24,25], while others suggest a decrease postmenopause [26], or an increase after typical menopausal age [27]. Similarly, studies from other regions suggest that cervical lesion rates may rise with menopause [28]. Notably, most prior studies have relied on age as a proxy for menopausal status, potentially obscuring the biological and clinical impacts of menopause itself. To date, no known studies have investigated this relationship in African women, despite the region’s disproportionately high burden of cervical cancer.

To address this critical gap in knowledge, we conducted a secondary data analysis of women aged 40–60 in Senegal. Our first objective was to examine whether menopausal status is associated with HPV detection, and our second objective was to examine whether menopausal status is associated with cervical neoplasia detection in this understudied population. By investigating these associations, this study aims to clarify the potential role of menopause in HPV detection and cervical lesion development, with the goal of informing screening and prevention strategies for older women in Senegal and other LMICs.

## Methods

### Overview

This study used retrospective data from three parent research studies conducted in Dakar, Senegal, between 1998 and 2010. Study participants included 3,180 women aged 40–60 with known menopausal status. We assessed associations between menopausal status and both HPV detection and cervical neoplasia detection. Additionally, we evaluated whether these associations varied by HPV risk subtype or cervical neoplasia grade, and whether Human Immunodeficiency Virus (HIV) status modified these associations.

### Study participants

The parent studies recruited women living in Senegal from three outpatient clinics: a primary care clinic (Centre de Santé Dominique de Pikine), an infectious disease clinic (CHNU-Fann, SMIT, Dakar), and an oncology clinic (Hôpital Aristide Le Dantec, Institute Curie, Dakar). Each parent study recruited participants from these clinics. The “Epidemiology and Biology of Cervical Neoplasia” parent study enrolled women aged 35 years and older from the Pikine primary care clinic and Dantec oncology clinic. The “New Approaches to Cervical Cancer Control” parent study enrolled women aged 18 years and older from the Fann infectious disease clinic and Dantec oncology clinic. The “DNA Hypermethylation in Cervical Cancer” parent study enrolled women aged 18 years and older from the Pikine primary care clinic and Fann infectious disease clinic. Aside from age and clinic-specific recruitment, there were no additional inclusion or exclusion criteria for enrollment in the parent studies. Some women attending the Dantec oncology clinic were referred with presumed cervical cancer; however, participants diagnosed with cervical cancer were excluded from the present secondary analysis. Women attending the Pikine primary care clinic and the Fann infectious disease clinic presented for routine clinical care unrelated to cancer. None of the participants included in our secondary analysis presented to these clinics for follow-up of previous cervical cancer screening or treatment at the time of enrollment in the parent studies.

For our secondary analysis using this data, we limited the study population to women aged 40–60 who had a satisfactory Pap smear and/or HPV test. This age range was selected because both menopausal and premenopausal women were represented within it, while nearly all women above 60 were postmenopausal. Restricting to ages 40–60 also reduced potential confounding by sexual activity patterns and aligned with the populations targeted in prior studies of menopause and HPV, as well as those for whom cervical cancer screening remains relevant.

**Fig 1:**
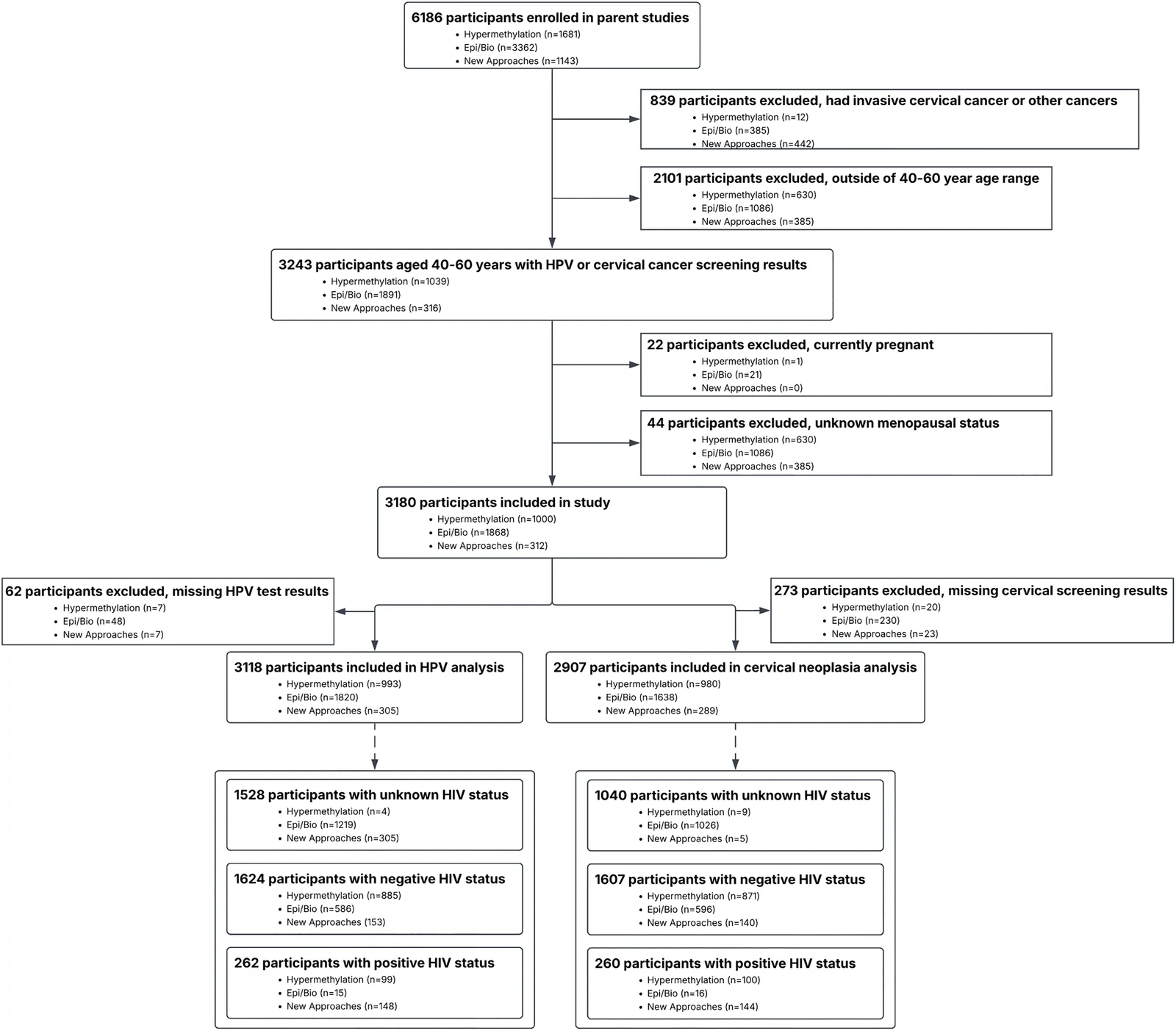
Participant selection from three parent studies. Abbreviations: DNA Hypermethylation in Cervical Cancer (Hypermethylation), Epidemiology and Biology of Cervical Neoplasia (Epi/Bio), and New Approaches to Cervical Cancer Control (New Approaches).

Participants were excluded if they had invasive cervical cancer (ICC), if they were currently pregnant, or if their menopausal status was unknown or could not be derived. Additional exclusions varied by analysis: for our first analysis, participants with an unsatisfactory HPV test were excluded, and for our second analysis, participants with an unsatisfactory cytology test were excluded (Fig 1).

### Study setting and design

The three parent studies, “Epidemiology and Biology of Cervical Neoplasia”, “New Approaches to Cervical Cancer Control”, and “DNA Hypermethylation in Cervical Cancer”, previously described in the literature, aimed to develop cervical cancer prevention strategies and identify biomarkers of HPV-related disease progression [29–32]. The parent studies, which enrolled study subjects February 18, 1998 to October 13, 2003, March 12, 2003 to December 19, 2006, and September 4, 2006 to October 13, 2010 respectively, were approved by the University of Washington and Senegalese Human Subjects Institutional Review Boards with written informed consent obtained from each participant. At study baseline, participants completed questionnaires collecting socio-demographic information, sexual and reproductive history, contraceptive use, and medical history. Data were de-identified and shared variables across studies were pooled for analysis. Data for this research was accessed on June 1, 2025 and is available as an excel file (S1 File).

Participants’ menopausal status was determined through questionnaire responses. Participants self-reported whether they had gone through menopause (“yes”, “no”, or “don’t know”) and provided the date of their last menstrual period (LMP). Participants were classified as postmenopausal if they answered “yes” to having gone through menopause. If menopausal data were missing or unknown (n = 308), status was imputed as postmenopausal if the LMP date was ≥ 12 months before study baseline. For participants missing both menopause and LMP data (n = 52), menopausal status that was gathered at a subsequent visit was used. If menopausal status could not be obtained, participants were excluded (n = 44).

Cervical swab samples collected at study baseline and follow up were tested for HPV DNA using polymerase chain reaction (PCR) assays using MY09/MY11 L1 consensus primers. Amplification of the cellular β-globulin gene served as a control. HPV DNA presence was determined by PCR amplification, followed by dot blot hybridization, and any positive samples were subsequently genotyped for type-specific HPV using either a Roche line blot (27 HPV types) or a Roche Linear Array assay (38 HPV types). For analysis, only a patient’s baseline cervical swab result was used to classify HPV status. High-risk HPV types included HPV-16, HPV-18, HPV-26, HPV-31, HPV-33, HPV-35, HPV-39, HPV-45, HPV-51, HPV-52, HPV-56, HPV-58, HPV-59, HPV-68, HPV-69, HPV-73, and HPV-82 while all others (HPV-6, HPV-11, HPV-40, HPV-42, HPV-53, HPV-54, HPV-55, HPV-57, HPV-61, HPV-62, HPV-64, HPV-66, HPV-67, HPV-70, HPV-83, HPV-84, HPV-71, HPV-72, HPV-81, IS39, and CP6108) were classified as low-risk [33].

Cervical neoplasia status was determined using cervical cytology and histologic biopsy results. In the parent studies, Pap smears were conducted at baseline and follow up. Cervical biopsies, depending on parent study protocol, were conducted to confirm disease presence histologically at study entry, during follow-up or exit, and/or when clinically indicated. Cytology results were classified as unsatisfactory, negative, atypical squamous cells of uncertain significance (ASCUS), low-grade squamous intraepithelial lesion (LSIL), high-grade squamous intraepithelial lesion (HSIL), carcinoma in situ (CIS), or ICC. Histologic biopsy results were classified as negative, reactive atypical changes, cervical intraepithelial neoplasia (CIN) grades I, II, or III, CIS, or ICC. Participants were considered cervical neoplasia-positive if they had LSIL, HSIL, CIN I/II/III, or CIS based on histologic biopsy, or Pap smear if histologic biopsy was not undertaken. For our analysis, only a patient’s baseline cervical cytology and histologic biopsy results were used.

HIV-1 and HIV-2 status was determined through serologic testing at baseline in all but one parent study. Participants with antibody-positive tests for either HIV-1 or HIV-2 were classified as HIV-positive. If HIV testing data were unavailable (n = 1,232), participants were classified as HIV-negative, given the low prevalence of HIV among adult women in Senegal (0.4%) [34], and the similarly low prevalence observed among those tested in the Pikine Clinic (2.7%), where the majority of missing data occurred.

### Statistical analysis

Data were analyzed using R software version 4.3.1 and SAS version 9.4. For our first analysis, we used multivariable logistic regression to assess the association between menopausal status (pre vs post) and any HPV detection. Unadjusted and adjusted odds ratios (ORs), 95% confidence intervals (CIs), and p-values were calculated, adjusting for the confounders of age, number of prior pregnancies, and HIV status. Age and number of prior pregnancies were treated as continuous variables in all models, while HIV status was treated as a binary variable.

Then, we used a generalized estimating equations (GEE) approach to examine the association between menopausal status and the likelihood of HPV infection, separately, for both high-risk and low-risk HPV types. Positive, untyped results were not included in these analysis. Our model controlled for age, number of prior pregnancies, and HIV status as confounders. A GEE model was implemented at the infection level, rather than the individual level, as each participant was tested for 27 or 38 HPV types concurrently, based on parent study assay type. The analysis was performed separately on subsetted data for high-risk and low-risk HPV infections. The logit link function with a binomial family was used to model the odds of HPV infection for each risk type. Specifically, the model compared the odds of detecting a given HPV type against the odds of not detecting that type, with no HPV detection serving as the reference category. An exchangeable correlation structure was specified to account for the correlation of repeated measurements within individuals. Robust standard errors were estimated for each risk type using the sandwich estimator. For our full first analysis, we also conducted a sensitivity analysis excluding participants with unknown HIV status. Additionally, multivariable logistic regression was used to assess the association between menopausal status and multiple HPV infections detected at once, as well as co-infection subcategories including high- and low-risk co-detection, high-risk only multiple infections, and low-risk only multiple infections, adjusting for the same confounders.

To evaluate whether the association between menopausal status and any HPV detection was modified by HIV status, we included an interaction term in our logistic regression model. Age and number of prior pregnancies were adjusted for, and ORs, 95% CIs, and p-values were reported. To further explore potential effect modification and confounding, we also conducted separate multivariable logistic regression models stratified by HIV status (negative and positive), adjusting for the same covariates.

For our second analysis, we used multivariable logistic regression to assess the association between menopausal status (pre vs post) and any cervical neoplasia detection. As in our first analysis, our model adjusted for age, number of prior pregnancies, and HIV status, and calculated ORs, 95% CIs, and p-values. To evaluate whether the association between menopausal status and any cervical neoplasia detection was modified by HIV status, we included an interaction term in our logistic regression model and performed the same separate multivariable logistic regression models as described above. Age and number of prior pregnancies were adjusted for, and ORs, 95% CIs, and p-values were reported.

Lastly, multinomial logistic regression was used to examine whether menopausal status was associated with differences in the detection of low- or high-grade cervical neoplasia. The same confounders were included in the model as in previous analyses and ORs, 95% CIs, and p-values were calculated. For our full second analysis, we also conducted a sensitivity analysis excluding participants with unknown HIV status.

## Results

### Demographic characteristics of the study population

Our study included 3,180 women aged 40–60 with a satisfactory Pap smear (N = 2,097) and/or HPV test (N = 3,118) who were able to be classified as premenopausal (n = 2,301, 72.4%) or postmenopausal (n = 879, 27.6%) (Table 1). Women pre and postmenopause were similar in most demographic factors. The vast majority of participants (> 97% overall) were born in Senegal with Wolof being the predominant ethnic group (~ 60% overall). More than half of the participants had no formal education and birth control use was infrequent (28.8% pre, 6.0% post), with injectables and condoms being most commonly used. Multiple pregnancies during a participant’s lifetime were very common, with roughly half of all women having between six and ten prior pregnancies. Over half the participants were in polygamous marriages, however, the lifetime number of sex partners was generally low, with the majority of women reporting only one partner (60.9% pre, 54.0% post), and fewer than 1% reporting more than five.

**Table 1:** Demographic, health, partnership, and reproductive characteristics among Senegalese women aged 40-60, categorized by self-reported menopausal status.

Characteristic	Menopausal Status	
	Premenopause *N = 2301*	Postmenopause *N = 879*
	*n (proportion %)*	*n (proportion %)*
Birthplace		
Senegal	1158 (97.6%)	574 (98.3%)
Other West Africa	24 (2.0%)	10 (1.7%)
Central Africa	2 (0.2%)	0 (0%)
Other	3 (0.3%)	0 (0%)
*Missing*	*1114 (48.4%)*	*295 (33.6%)*
Ethnic Group		
Wolof	1159 (58.5%)	512 (62.9%)
Pulaar	356 (18.0%)	124 (15.2%)
Serere	186 (9.4%)	84 (10.3%)
Sarakhole	28 (1.4%)	7 (0.9%)
Mandjack	26 (1.3%)	13 (1.6%)
Diola	57 (2.9%)	20 (2.5%)
Bambara	36 (1.8%)	8 (1.0%)
Other	133 (6.7%)	46 (5.7%)
*Missing*	*320 (13.9%)*	*65 (7.4%)*
Age Categories (Years)		
40-45	1486 (64.6%)	125 (14.2%)
46-50	648 (28.2%)	281 (32.0%)
51-55	144 (6.3%)	275 (31.3%)
56-60	23 (1.0%)	199 (22.5%)
Education Level		
None	710 (52.7%)	451 (67.0%)
Primary	395 (29.3%)	157 (23.3%)
Secondary	225 (16.7%)	60 (8.9%)
University	16 (1.2%)	5 (0.7%)
*Missing*	*955 (41.5%)*	*206 (23.4%)*
Marital Status		
Single	18 (0.8%)	8 (0.9%)
Married – monogynous	802 (34.9%)	202 (23.2%)
Married – polygamous	1200 (52.3%)	472 (54.2%)
Separated/Divorced	150 (6.5%)	74 (8.5%)
Widowed	121 (5.3%)	115 (13.2%)
Concubine	2 (0.1%)	0 (0%)
Number of Cowives (only for married – polygamous participants)		
1	359 (57.3%)	166 (49.7%)
2	176 (28.1%)	96 (28.7%)
3	78 (12.5%)	51 (15.3%)
4+	13 (2.1%)	21 (15.3%)
*Missing*	*574 (47.8%)*	*138 (29.2%)*
Birth Control/Contraception Method		
None	1634 (71.2%)	823 (93.9%)
Pill	11 (0.5%)	4 (0.5%)
Injection	182 (7.9%)	10 (1.1%)
Condoms	194 (8.5%)	8 (0.9%)
Spermicide	1 (0.04%)	0 (0%)
IUD	3 (0.1%)	0 (0%)
Other	269 (11.7%)	31 (3.5%)
Age of Last Pregnancy (Years)		
11-20	28 (2.5%)	18 (3.3%)
21-40	855 (76.1%)	382 (69.1%)
41-60	241 (21.4%)	151 (27.7%)
*Missing*	*1177 (51.2%)*	*326 (37.1%)*
Number of Pregnancies		
0	47 (2.0%)	23 (2.6%)
1-5	592 (25.7%)	197 (22.4%)
6-10	1256 (54.6%)	452 (51.4%)
11-20	404 (17.6%)	207 (23.5%)
Lifetime Number of Sex Partners		
1	1126 (60.9%)	415 (54.0%)
2-5	704 (38.1%)	348 (45.3%)
6-10	15 (0.8%)	3 (0.4%)
>10	4 (0.2%)	2 (0.3%)
*Missing*	*452 (19.6%)*	*111 (12.6%)*
HIV Status		
Negative	1111 (87.0%)	543 (84.1%)
HIV-1	119 (9.3%)	69 (10.7%)
HIV-2	37 (2.9%)	24 (3.7%)
Both HIV-1 & HIV-2	10 (0.8%)	10 (1.5%)
*Missing*	*1024 (44.5%)*	*233 (26.5%)*
Smoking Status		
Never	1166 (98.5%)	577 (98.8%)
Current	14 (1.2%)	5 (0.9%)
Past	4 (0.3%)	2 (0.3%)
*Missing*	*1117 (48.5%)*	*295 (33.6%)*

Missing values that exceed 1% are presented as unweighted percentages.

Substantial missingness was observed for some variables. Missing data on HIV status, age of last pregnancy, birthplace, education level, number of cowives for participants in a polygamous marriage, and current smoking status was high across participants (40.0%, 47.3%, 44.3%, 36.5%, 42.6%, and 44.4% overall, respectively). Missing data can be attributed to the fact that all participants in the parent studies attended an initial study visit, but only a portion of them attended a subsequent study visit, which collected more demographic, behavioral, and health history data than screening alone.

### Menopause and HPV detection

Overall, 870 of 3,118 participants (27.9%) tested positive for HPV, with a higher proportion of cases being postmenopausal (24.5% pre, 36.6% post) (Table 2). The association between menopause and HPV detection was similar in HIV-negative (OR = 1.48; CI = 1.17, 1.87) and HIV-positive women (OR = 1.42; CI = 0.67, 3.09) (data not reported in tables), and no evidence of interaction was observed between menopausal status and HIV status on HPV detection (*p* = 0.78).

**Table 2:** Association between menopausal status and HPV detection in Senegalese women aged 40-60.

Outcome	Unadjusted-Premenopause N = 2,253 n (proportion %)	Unadjusted-Postmenopause N = 865 n (proportion %)	Unadjusted Odds Ratio (95% CI)	Adjusted* Odds Ratio (95% CI)
Any HPV Infection Detected (Low- or High-risk):	553 (24.5%)	317 (36.6%)	1.78 (1.50, 2.10)	1.48 (1.18, 1.85)
Multiple HPV Infections Detected:	187 (8.3%)	158 (18.3%)	2.47 (1.97, 3.10)	1.94 (1.40, 2.69)
Pattern of Multiple HPV Infections Detected:				
High- & Low-Risk HPV Co-Detected:	128 (5.7%)	122 (14.1%)	2.73 (2.10, 3.54)	2.12 (1.48, 3.04)
Only High-Risk HPV Co-Detected:	40 (1.8%)	16 (1.8%)	1.04 (0.56, 1.84)	0.97 (0.43, 2.05)
Only Low-Risk HPV Co-Detected:	19 (0.8%)	20 (2.3%)	2.78 (1.47, 5.28)	1.63 (0.71, 3.68)

*Adjusted for age, number of prior pregnancies, and HIV status at the participant level.

However, HIV status was identified as a potential confounder and was therefore included in all adjusted models. After adjustment for age, number of prior pregnancies, and HIV status, postmenopausal women had 1.48 times higher odds of HPV detection compared to premenopausal women (OR = 1.48; CI = 1.18, 1.85). Postmenopausal women also had significantly higher odds of multiple HPV infections compared to premenopausal women, with a stronger magnitude of association than for any HPV detection (OR = 1.94; CI: 1.40, 2.69), after adjustment for confounders. When examining co-infection patterns, most women had both high-risk and low-risk HPV infections detected, with postmenopausal women (14.1%) compared to premenopausal women (5.7%), having more than twice the odds of high-risk and low-risk co-infection (OR = 2.12; CI: 1.48, 3.04), after adjustment for confounders. The subcategories of high-risk only and low-risk only multiple infections had limited samples sizes (n = 56 and n = 39, respectively) and menopausal status was not significantly associated with co-infection with these patterns.

Of the 1,671 HPV infections identified, 1,529 (91.5%) were successfully typed (S1 Table). The most common types detected were HPV-54 (n = 100, 6.5%), HPV-16 (n = 96, 6.3%), HPV-52 (n = 95, 6.2%), HPV-53 (n = 92, 6.0%), and HPV-58 (n = 91, 6.0%). Of those, HPV types 16, 52, and 58 are high-risk. The distribution of high-risk and low-risk HPV infections among women with HPV was similar (~53% and 47%, respectively), among the two menopausal groups. However, the odds of infection differed significantly by menopausal status.

In GEE models, postmenopausal women had 1.64 times higher odds of low-risk HPV infection compared to premenopausal women (OR = 1.64, CI = 1.30, 2.08) (Table 3) and 1.55 times higher odds of high-risk HPV infection (OR = 1.55, CI = 1.25, 1.93) (Table 4), after adjusting for age, number of prior pregnancies, and HIV status. Results from the sensitivity analysis excluding participants with unknown HIV status were consistent with the primary analysis. Postmenopausal women had similar odds of any HPV detection (OR = 1.51; CI: 1.17, 1.95), low-risk HPV detection (OR = 1.56; CI: 1.23, 1.97), and high-risk HPV detection (OR = 1.55; CI: 1.25, 1.92) (data not reported in tables).

**Table 3:** Association between menopausal status and low-risk HPV detection in Senegalese women aged 40-60.

Outcome	Unadjusted Odds Ratio* (95% CI)	Adjusted** Odds Ratio* (95% CI)
No HPV Detected:	—	—
Low-Risk HPV Detected:	1.98 (1.58, 2.48)	1.64 (1.30, 2.08)

*ORs calculated using GEE analysis

**Adjusted for age, number of prior pregnancies, and HIV status at the participant level.

**Table 4:** Association between menopausal status and high-risk HPV detection in Senegalese women aged 40-60.

Outcome	Unadjusted Odds Ratio* (95% CI)	Adjusted** Odds Ratio* (95% CI)
No HPV Detected:	—	—
High-Risk HPV Detected:	1.69 (1.38, 2.08)	1.55 (1.25, 1.93)

*ORs calculated using GEE analysis

**Adjusted for age, number of prior pregnancies, and HIV status at the participant level.

### Menopause and cervical neoplasia detection

Overall, 287 of 2,907 participants with valid cytology and/or histology were classified as having any grade of cervical neoplasia, with a higher proportion being postmenopausal (9.2% pre, 11.7% post) (Table 5). After adjustment for age, number of prior pregnancies, and HIV status, postmenopausal women had 1.36 times higher odds of cervical neoplasia detection compared to premenopausal women (OR = 1.36; CI = 0.97, 1.90), however this result was not statistically significant. Furthermore, after adjustment, the association between menopause and any cervical neoplasia detection differed between HIV-negative (OR = 1.72; CI = 1.16, 2.52) and HIV-positive women (OR = 0.76; CI = 0.39, 1.44), with only the estimate for HIV-negative women being statistically significant (*p* = 0.006) (data not reported in tables). Importantly, the formal test for interaction was not statistically significant (*p* = 0.14), indicating no substantial evidence that HIV status modifies the association between menopausal status and cervical neoplasia detection.

**Table 5:** Association between menopausal status and detection of low- and high- grade cervical neoplasia in Senegalese women aged 40-60.

Outcome	Unadjusted-Premenopause N = 2,095 n (proportion %)	Unadjusted-Postmenopause N = 812 n (proportion %)	Unadjusted Odds Ratio (95% CI)	Adjusted* Odds Ratio (95% CI)
No Cervical Neoplasia Detected:	1,903 (90.8%)	717 (88.3%)	—	—
Any Cervical Neoplasia Detected^**^:	192 (9.2%)	95 (11.7%)	1.31 (1.01, 1.70)	1.36 (0.97, 1.90)
Low Grade^†^ Cervical Neoplasia Detected:	100 (4.8%)	51 (6.3%)	1.35 (0.96, 1.92)	1.30 (0.84, 2.02)
High Grade^††^ Cervical Neoplasia Detected:	92 (4.4%)	44 (5.4%)	1.27 (0.88, 1.84)	1.43 (0.90, 2.28)

*Adjusted for age, number of prior pregnancies, and HIV status at the participant level.

**Low grade or worse: LSIL or CIN1, HSIL or CIN2–3 or CIS.

†LSIL or CIN1.

††HSIL or CIN2 or CIN3 or CIS.

Of 287 cervical neoplasia cases, 52.6% were low grade, and 47.4% were high grade. Among premenopausal women, 4.4% (n = 92) had high-grade cervical neoplasia detected, compared to 5.4% (n = 44) of postmenopausal women (Table 5). After adjusting for age, number of prior pregnancies, and HIV status, postmenopausal women had slightly higher, but not statistically significant, odds of low-grade cervical neoplasia detection compared to premenopausal women (OR = 1.30; CI = 0.84, 2.02). Similarly, postmenopausal women had somewhat higher, but not statistically significant, odds of high-grade cervical neoplasia detection (OR = 1.43; CI = 0.90, 2.28). In the sensitivity analysis excluding participants with unknown HIV status, the associations between menopausal status and cervical neoplasia were attenuated compared to the primary analysis. Postmenopausal women had slightly higher, but non-significant, odds of low-grade (OR = 1.18; CI: 0.75, 1.85) and high-grade cervical neoplasia (OR = 1.16; CI: 0.72, 1.86) compared to premenopausal women. In stratified analyses among HIV-negative participants with known status, odds of low-grade (OR = 1.42; CI: 0.82, 2.45) and high-grade cervical neoplasia (OR = 1.31; CI: 0.80, 2.46) remained elevated but did not reach statistical significance (data not reported in tables). Among HIV-positive participants, odds of both low-grade (OR = 0.80; CI: 0.36, 1.76) and high-grade cervical neoplasia (OR = 0.70; CI: 0.29, 1.73) were not significantly associated with menopausal status (data not reported in tables).

## Discussion

In this study of Senegalese women aged 40–60, postmenopausal women had significantly higher odds of HPV detection compared to premenopausal women, even after adjusting for age, number of prior pregnancies, and HIV status. This also held true when examining low-risk and high-risk HPV types separately, where the odds reflect the likelihood of detecting each individual HPV type compared to not detecting that type. Postmenopausal women also had somewhat higher odds of cervical neoplasia detection overall, and this held true for both low-grade and high-grade neoplasia, although these estimates were not statistically significant. Our findings support the hypothesis that menopause is associated with HPV persistence, reactivation, and potentially the development of cervical lesions [21,23], and can offer explanation as to why HPV prevalence in women often displays a bimodal distribution with a secondary peak later in life [15–18]. Notably, the association with menopausal status was strongest for multiple HPV infections, and particularly for high- and low-risk co-infections. These results are consistent with the hypothesis that immune decline associated with menopause may impair clearance of concurrent HPV infections [21,23]. Mixed high- and low-risk co-infections may increase the potential for cervical disease progression, as the presence of high-risk types, regardless of co-infection status, carries oncogenic risk that warrants clinical attention. Results from our sensitivity analysis excluding participants with unknown HIV status were consistent with our primary findings for HPV detection, supporting the validity of classifying participants with missing HIV data as HIV-negative for this analysis.

The relationship between menopausal status and cervical neoplasia detection was less clear and was further nuanced by HIV status. While the formal test for interaction between menopausal status and HIV status was not statistically significant (p = 0.14), stratified analyses suggested that the association between menopause and cervical neoplasia may be more pronounced among HIV-negative women than among HIV-positive women. This pattern was further supported by our sensitivity analysis excluding participants with unknown HIV status, rather than imputing those individuals to being HIV-negative. In the sensitivity analysis, overall associations between menopausal status and both low-grade and high-grade cervical neoplasia attenuated compared to the primary analysis. This attenuation may reflect the fact that participants with missing HIV data were disproportionately from the Pikine primary care clinic, where HIV prevalence among tested individuals was low (2.7%). However, if HIV-positive individuals were overrepresented among those with missing data, classifying them as HIV-negative in the primary analyses could have introduced residual confounding, potentially inflating the observed association between menopausal status and cervical neoplasia. Among HIV-negative women with known status, the odds of both low-grade and high-grade cervical neoplasia remained elevated, though these grade-specific estimates did not reach statistical significance, likely reflecting smaller sample sizes within HIV-status strata for these less common outcomes. It should also be noted that our study was not powered for stratified analysis by HIV status, and these stratum-specific estimates should therefore be interpreted with caution. This elevation may suggest the association between menopause and cervical neoplasia is particularly relevant among HIV-negative women. Among HIV-positive women, immune suppression related to HIV infection itself may be more impactful than immunologic changes related to menopause.

To the best of our knowledge, our study is the first to show postmenopausal women having a statistically significant increased odds of HPV infection compared to premenopausal women, along with potentially higher observed odds of cervical neoplasia detection. Few studies have directly compared HPV or cervical neoplasia prevalence between pre and postmenopausal women, and most findings have differed from ours. For example, a study examining women in Wuhan, China found no significant difference between HPV infection rates in postmenopausal and premenopausal women (15.1% and 12.3%, respectively; *p* = 0.056) [22]. However, in that study, menopausal status was inferred based on age rather than clinical criteria, which may have led to misclassification of menopausal status. Similarly, another study from China reported comparable HPV, cervical neoplasia, and cervical cancer prevalence across menopausal groups (*p* > 0.05) [25]. That study determined menopausal status using a combination of self-report and age-based imputation, with over 20% of the postmenopausal group classified based on an age-range cut off rather than biological criteria. This likely introduced exposure misclassification, which could have hidden true differences between groups. In contrast, our study adjusted for age in all models and used both self-reported menopausal status and LMP data to classify exposure.

A study from the United Arab Emirates that did adjust for age found high-risk HPV infection and progression from ASCUS lesions to high-grade lesions to be more common in premenopausal women (20.7% and 28.7%, respectively) compared to postmenopausal women (9.5% and 12.8%, respectively) [26], which contrasts with our findings. This discrepancy may reflect behavioral or healthcare system differences between the study populations, or differences in the underlying prevalence, persistence, and reactivation of high-risk HPV types geographically. Interestingly, one US-based study that adjusted for age, among other confounders, found that perimenopausal women had significantly higher HPV prevalence than premenopausal women. Postmenopausal women also had elevated HPV prevalence, though this was not statistically significant [27]. Their findings suggest a possible increase in HPV prevalence postmenopause, but the study was underpowered and did not detect a statistically significant association, given the small sample size (N = 172). Our study differed in that we dichotomized menopausal status, rather than including a perimenopausal category. This difference, as well as our study having a larger sample size, and therefore sufficient power to detect a significant association, may explain the difference in conclusions.

Although few studies have examined HPV burden within postmenopausal populations, one earlier longitudinal study in the United States found that over one-third of postmenopausal women tested positive for HPV at least once over a seven-year period, with two-thirds of infections involving high-risk oncogenic types [35]. These findings, as well as our own, highlight that postmenopausal women have a high burden of HPV and show a trend toward higher prevalence of pre-invasive disease, which could develop into cancer if left undetected and untreated. Notably, this elevated HPV burden post menopause is unlikely to reflect new sexual exposures, as in younger women, as women around menopausal age tend to report infrequent new sexual partnerships [36]. Our findings support the hypothesis that these infections likely represent the persistence or reactivation of latent HPV acquired earlier in life, potentially triggered by hormonal and immune changes associated with menopause [15].

Our study has several strengths. To date, it is one of the largest studies to assess the association between menopausal status and both HPV and cervical neoplasia. Importantly, our study evaluated both high-risk and low-risk HPV types together and separately, using both Pap smear and biopsy data to assess disease. We also adjusted for potential confounding by age, a step that was missing or inconsistently applied across prior studies, and restricted our sample to women in the age range where menopausal transition typically occurs. Furthermore, we classified menopausal status using self-report and LMP dates to minimize misclassification, rather than using age as a proxy to menopause. Our findings are particularly valuable given that the study population was unscreened, providing insight into HPV and cervical disease burden in a setting more reflective of many LMICs, where under-screening and low vaccination coverage are still the reality. Although our data were collected prior to HPV vaccination introduction, vaccination rates among Senegalese women aged 40–60 remain low [12], suggesting that our findings remain relevant.

Despite the strengths of our study, several limitations should be considered when interpreting the findings. First, generalizability may be limited due to the unique behavioral, health, and cultural characteristics of our cohort, including the prevalence of polygamous marriage, fewer sexual partners in a lifetime, higher parity, and lower usage of hormonal contraception [36], all of which may influence HPV exposure dynamics compared to other populations [37,38]. Immune function may differ in this setting due to the higher prevalence of infectious diseases and malnutrition, even among HIV-negative individuals [39]. Menopausal status was determined based on status self-report and LMP, which may lead to non-differential misclassification if participants were unaware of the definition of menopause, or if conditions such as malnutrition or chronic stress caused loss of menstruation unrelated to menopause [40]. Such misclassification could affect the accuracy of our exposure classification and may have impacted the observed associations between menopausal status and HPV and cervical neoplasia detection. HPV genotyping also relied on two different assays across the parent studies, a Roche Line Blot detecting 27 HPV types and a Roche Linear Array detecting 38 types, which may have led to underdetection of certain HPV types among participants tested with the Line Blot. Further, findings from our research assays may not be generalizable to HPV DNA or mRNA assays currently approved for primary HPV screening. Additionally, physiological changes in the cervix after menopause may have created challenges for cervical lesion detection [41,42], potentially leading to underestimation of the true effect of menopause on cervical neoplasia. Additionally, while sensitivity analyses excluding participants with unknown HIV status were conducted, the attenuation of cervical neoplasia associations observed in these analyses suggests that residual confounding by HIV status cannot be ruled out in our primary cervical neoplasia estimates, and these findings should be interpreted with this limitation in mind. Finally, cervical neoplasia was relatively uncommon in our study population, which limited statistical power for some analyses. The elevated but non-significant odds ratios observed should therefore be interpreted cautiously, and larger studies are needed to confirm these associations.

These limitations do not diminish the clinical and public health relevance of our findings, particularly given the magnitude and consistency of the observed associations. While low-risk HPV types and low-grade cervical neoplasia are common, they are often transient and can resolve without intervention due to effective cell-mediated immune clearance [43]. Low-risk types rarely integrate into host DNA or lead to high-grade disease [43,44]. In contrast, high-risk HPV types are more likely to persist, potentially with immune decline post menopause, and can lead to integration into host genetic material, an important step in cervical carcinogenesis [43,44]. This distinction is critical, as it explains why the increased detection of high-risk HPV and high-grade neoplasia among postmenopausal women in our study is particularly clinically relevant, more so than the increase in low-risk infections or low-grade lesions alone.

Our findings challenge the assumption that HPV is primarily a concern for younger women and highlight the need for continued surveillance and screening among older women, even those perceived to be at a lower risk of infection. Our observed increased detection of high-risk oncogenic HPV among postmenopausal women highlights their vulnerability to future cervical cancer development and reinforces the importance of screening strategies that identify infections early, before cancer can develop. We also observed a higher, though non-significant, burden of cervical lesions in postmenopausal women, consistent with a trend toward increased risk of HPV disease progression. However, screening and treatment for cervical neoplasia in postmenopausal women poses unique clinical challenges. For example, physiological changes such as atrophy of the cervix and the presence of a type three transformation zone can make it more difficult to visualize and sample the cervix, potentially leading to missed lesions [41,42]. Due to these limitations, it is possible that the true burden of cervical neoplasia in postmenopausal women may be greater than we observed. Furthermore, given that there is a long latency period between persistent HPV infection and cervical cancer onset [45], and the possibility of viral reactivation after menopause [15], improving screening strategies to identify high-risk infections and cervical neoplasia in postmenopausal women is critical. Given the limitations of cytology in postmenopausal women, primary high-risk HPV testing may be more accurate for screening postmenopausal women for cervical cancer [46]. Broader implementation of primary high-risk HPV screening presents an important opportunity for intervention and cancer prevention in this population. While precise estimates of the proportion of ICC cases diagnosed among postmenopausal women in Sub-Saharan Africa are unavailable, evidence suggests that a substantial fraction (85%) of ICC burden worldwide is placed on this region [47]. Strengthening surveillance systems that capture age-specific and menopausal status data will assist in quantifying the burden and inform age-tailored screening policies.

For women living without HIV, the World Health Organization recommends HPV-based cervical cancer screening every five to ten years in women beginning at age 30, with an upper age limit of 65 for those who have been regularly screened and consistently tested negative [48]. These guidelines prioritize screening among women <49 years and secondarily, those aged 50–65 [48]. Additionally, in Senegal, and many other LMICs, HPV vaccination and cervical cancer screening coverage and access remain very low [12]. In this context, screening is not only essential, but it might be the only opportunity for early detection and prevention of cervical cancer in most women. Given our findings that postmenopausal women in Senegal had significantly higher odds of high-risk HPV infection and high-grade cervical neoplasia, expanding screening to include postmenopausal women, especially those who had never been screened, may be critical in LMICs to help prevent future cervical cancer cases, regardless of age. Finally, while prior research has established several risk factors for HPV infection, including a high number of lifetime sex partners, oral contraceptive use, age, altered immune status, and cigarette smoking [1,37], our findings suggest that menopause may also play a role as a risk factor for HPV persistence, potential reactivation, and the development of cervical lesions. Future longitudinal research, especially in African populations at highest risk for development of invasive cervical diseases, should focus on the association between all phases of menopause, including time since onset of menopause, and HPV infection risk, to better understand how the timing and progression of menopause influence the relationship between menopause and HPV.

## Supporting information

S1 Table Distribution of HPV infection counts by type and menopausal status in Senegalese women aged 40–60. (DOCX)

S1 File Excel file of data related to this analysis. (XLSX)
