# Supplementary material for: Impact of menopausal status on human papillomavirus detection and cervical neoplasia in middle-aged Senegalese women
Source: PLoS One. 2026 Sep 11;21(9):e0358080. doi: 10.1371/journal.pone.0358080 (PMC13567829; doi:10.1371/journal.pone.0358080)
Supplement: Supplementary file 1 [file pone.0358080.s001.docx]

**S1 Table. Distribution of HPV infection counts by type and menopausal status in Senegalese women aged 40-60.**

| HPV Type Detected | **Premenopause**  N Infections = 965 N Participants = 2,253  **______________** n (proportion of infections, %)* | **_____________** n (infections per 100 participants) | **Postmenopause**  N Infections = 706 N Participants = 865  **_______________** n (proportion of infections, %)* | **_____________** n (infections per 100 participants) |
| --- | --- | --- | --- | --- |
| *Low-Risk* |  |  |  |  |
| 6 | 15 (1.7%) | 0.7 | 8 (1.2%) | 0.9 |
| 11 | 3 (0.3%) | 0.1 | 5 (0.8%) | 0.6 |
| 40 | 5 (0.6%) | 0.2 | 5 (0.8%) | 0.6 |
| 42 | 14 (1.6%) | 0.6 | 12 (1.8%) | 1.4 |
| 53 | 53 (6.1%) | 2.4 | 39 (5.9%) | 4.5 |
| 54 | 58 (6.7%) | 2.6 | 42 (6.3%) | 4.9 |
| 55 | 11 (1.3%) | 0.5 | 9 (1.4%) | 1.0 |
| 57 | 0 (0.0%) | 0.0 | 0 (0.0%) | 0.0 |
| 61 | 45 (5.2%) | 2.0 | 45 (6.8%) | 5.2 |
| 62 | 30 (3.5%) | 1.3 | 31 (4.7%) | 3.6 |
| 64 | 1 (0.1%) | 0.0 | 1 (0.2%) | 0.1 |
| 66 | 23 (2.7%) | 1.0 | 14 (2.1%) | 1.6 |
| 67 | 2 (0.2%) | 0.1 | 2 (0.3%) | 0.2 |
| 70 | 16 (1.9%) | 0.7 | 10 (1.5%) | 1.2 |
| 71 | 11 (1.3%) | 0.5 | 12 (1.8%) | 1.4 |
| 72 | 6 (0.7%) | 0.3 | 7 (1.1%) | 0.8 |
| 81 | 26 (3.0%) | 1.2 | 26 (3.9%) | 3.0 |
| 83 | 43 (5.0%) | 1.9 | 34 (5.1%) | 3.9 |
| 84 | 19 (2.2%) | 0.8 | 19 (2.9%) | 2.2 |
| IS39 | 2 (0.2%) | 0.1 | 4 (0.6%) % | 0.5 |
| CP6108 | 5 (0.6%) | 0.2 | 14 (2.1%) | 1.6 |
| *Low-Risk Total* | *388 (44.9%)* | *17.2* | *339 (51.0%)* | *39.2* |
| *High-Risk* |  |  |  |  |
| 16 | 54 (6.2%) | 2.4 | 42 (6.3%) | 4.9 |
| 18 | 34 (3.9%) | 1.5 | 34 (5.1%) | 3.9 |
| 26 | 0 (0.0%) | 0.0 | 2 (0.3%) | 0.2 |
| 31 | 32 (3.7%) | 1.4 | 25 (3.8%) | 2.9 |
| 33 | 48 (5.6%) | 2.1 | 24 (3.6%) | 2.8 |
| 35 | 26 (3.0%) | 1.2 | 12 (1.8%) | 1.4 |
| 39 | 14 (1.6%) | 0.6 | 12 (1.8%) | 1.4 |
| 45 | 24 (2.8%) | 1.1 | 21 (3.2%) | 2.4 |
| 51 | 34 (3.9%) | 1.5 | 24 (3.6%) | 2.8 |
| 52 | 63 (7.3%) | 2.8 | 32 (4.8%) | 3.7 |
| 56 | 24 (2.8%) | 1.1 | 10 (1.5%) | 1.2 |
| 58 | 50 (5.8%) | 2.2 | 41 (6.2%) | 4.7 |
| 59 | 20 (2.3%) | 0.9 | 14 (2.1%) | 1.6 |
| 68 | 19 (2.2%) | 0.8 | 12 (1.8%) | 1.4 |
| 69 | 3 (0.3%) | 0.1 | 0 (0.0%) | 0 |
| 73 | 23 (2.7%) | 1.0 | 15 (2.3%) | 1.7 |
| 82 | 8 (0.9%) | 0.4 | 6 (0.9%) | 0.7 |
| *High-Risk Total* | *476 (55.1%)* | *21.1* | *326 (49.0%)* | *37.7* |
| Untyped | 101 (11.7%) | 4.5 | 41 (6.2%) | 4.7 |

*Counts represent the number of HPV infections detected (by type) within that menopausal group (pre or post), not the number of infected individuals. Some individuals had multiple HPV infections of different types.
