# Supplementary material for: Impact of menopausal status on human papillomavirus detection and cervical neoplasia in middle-aged Senegalese women
Source: PLoS One. 2026 Sep 11;21(9):e0358080. doi: 10.1371/journal.pone.0358080 (PMC13567829; doi:10.1371/journal.pone.0358080)
Supplement: Supplementary file 2 [file pone.0358080.s004.docx]

Dear Reviewers,

We sincerely thank you for your careful evaluation of our manuscript and for their thoughtful and constructive comments. We appreciate the time and effort invested in providing feedback, which has helped us improve the quality and clarity of the manuscript. We have carefully considered each comment and revised the manuscript accordingly. Below, we provide a point-by-point response to each reviewer comment.

**________________________________________________________________________**

**REVIEWER 1**

***Materials and Methods Section***

**Comment 1:**
The manuscript states that women from three studies were included. However, it is not clearly specified, for each study:

- which groups of women were included
- how many were excluded
- and the reasons for exclusion or inclusion
- It should also be clarified, for each group, how many HIV-positive and HIV-negative women were included.

A flowchart should be provided to visualize these data.

Flow chart examples:
 o Risk factors of human papillomavirus-related cervical lesions in postmenopausal women: a cross-sectional study (BMC Women’s Health, Springer Nature)

o Global prevalence of cervical human papillomavirus in women aged 50 years and older with normal cytology: a systematic review and meta-analysis (The Lancet Microbe).

**Response:**

Thank-you for this suggestion and for providing these two examples. A flow chart (Fig 1) has been added to the submission to illustrate participant selection from each of the three parent studies, including the groups of women included, the numbers and reasons for exclusion, and the numbers of HIV-positive, HIV-negative, and HIV-unknown women included in each of our two primary analyses. A figure title and caption have also been added to the manuscript. Please see the information below.

Below is the Figure which will be included:

**Comment 2:**
Inclusion Criteria: The criteria under which women were included in the previous studies should be clarified. Were the same criteria applied, and are the populations comparable? The authors do not specify whether women with precancerous results or those who had received prior treatments were included—data that could bias the results.

(similar to the ‘and the reasons for exclusion or inclusion above)

**Response:**

Inclusion criteria for the three parent studies were as follows: The “Epidemiology and Biology of Cervical Neoplasia” study enrolled women aged 35 and older from two study clinics: 1 - a primary care clinic (Centre de Santé Dominique de Pikine) and 2 - an oncology clinic (Hôpital Aristide Le Dantec, Institute Curie, Dakar). The “New Approaches to Cervical Cancer Control” study enrolled women aged 18 and older from two study clinics: 1 - an infectious disease clinic (CHNU-Fann, SMIT, Dakar) and 2 - 2 - an oncology clinic (Hôpital Aristide Le Dantec, Institute Curie, Dakar). Finally, the “DNA Hypermethylation in Cervical Cancer” study enrolled women 18 and older from two study clinics: 1 - a primary care clinic (Centre de Santé Dominique de Pikine) and 2 - an infectious disease clinic (CHNU-Fann, SMIT, Dakar). There were no further inclusion or exclusion criteria for entry into the parent studies. Women enrolling at the Dantec oncology clinic were referred to that clinic with presumed cancer (and if they had cancer, were excluded from the present secondary data analysis). Women enrolling at Pikine or Fann presented for various other reasons not related to cervical cancer and outside of our study, cervical cancer screening was not offered at those clinics. As cervical cancer screening is rare in Senegal, it is possible that some women were previous screened and/or treated for prior cervical lesions, but none presented for follow-up of cervical screening and/or treatment at the time of enrollment into our studies. We have added additional information to reflect the study eligibility criteria for the three parent studies to the methods section.

**Comment 3:**
Definition of Postmenopause: The definition of postmenopause should be more precise. Although data on the last menstrual period were available, this information was not consistently used and was only considered when not otherwise reported. If such data are available, they should be applied as a criterion to define women in the postmenopausal group.

**Response:**

Thank-you for this suggestion. As noted in the methods section, Information on whether a women was postmenopausal or not was obtained from self-report during the baseline study interview and was used as the primary determination of menopausal status. Information regarding last menstrual period (LMP) was also obtained during the interview and was used only when self-reported menopausal status was answered as “don’t know” or missing as LMP was also self-reported, seemed less reliable (often only month or year was known), and was often missing. If a woman was enrolled into follow-up, missing menopausal status was obtained from a future visit (n=52). Participants were excluded if menopausal status could not be obtained from these three sources (n=44). Information regarding the number of women excluded for these reasons combined has been included in Figure 1.

**Comment 4:**
Secondary Data: HPV and HIV detection, as well as cytology and histopathology results, were secondary data. Therefore, all groups should be detailed in a flow chart within the Materials and Methods section to facilitate comprehension.

**Response:**

Cervical samples were obtained for cytology and HPV detection in all study protocols. However, as some samples were inadequate for cytologic evaluation and/or HPV DNA testing, participants were excluded from analyses. Information on these exclusions and those with missing HIV testing is now included in the flow chart of study participants (new Figure 1).

***Results Section*

Comment 5:**
Table 1 should be expanded to include characteristics stratified by menopausal status, incorporating cytology and histology results by group, as well as HIV and HPV detection results. This is essential to visualize the number and proportion of women with these characteristics in each group.

**Response:**

We appreciate this suggestion by the reviewer. HIV status was already in Table 1 and remains reported there in our revision. However, as HPV and cervical abnormalities are the outcomes of interest, we have reserved reporting of those outcomes as before, in later tables. HPV overall (Table 2) and by specific type and grouped by low- and high-risk types (Table 3) are clearly presented, stratified by menopausal status. We feel that adding all of this information to Table 1 would be unwieldy and would result in a Table 1 beyond what is typical, covering multiple pages. Similarly, in Table 4, we present the combined cytology/histology results, again stratified by menopausal status. Please note that Tables have been renumbered in our revised manuscript.

**Comment 6:**
HIV Status Bias: A major bias is that HIV status information is missing for a large group of women. The authors mention that prevalence in Senegal is low (0.4%); however, significant associations with cervical neoplasia detection are observed only in HIV-negative women. Analyses should therefore separate HIV-positive and HIV-negative groups, excluding women with missing HIV data.

**Response:**

Thank you for sharing your concerns about our approach to imputing HIV status as negative in the subset of subjects on whom HIV testing was not conducted. HIV was considered a confounder in analyses related to HPV but was found to be a potential effect modifier in the association between menopausal status and cervical neoplasia. To address this concern, we conducted sensitivity analyses excluding participants with unknown HIV status for both our HPV and cervical neoplasia analyses, and have added the results of these sensitivity analyses to the results section. With regards to HPV detection, the sensitivity analysis results were consistent with our primary findings. The OR for any HPV detection associated with menopausal status changed minimally from 1.48 to 1.51 when participants with unknown HIV status were excluded, and results for both high-risk and low-risk HPV types were similarly unchanged. We therefore continue to present the primary HPV analyses using the imputed data in the tables, with sensitivity analysis results reported in the text. With regards to cervical neoplasia, the sensitivity analysis excluding participants with unknown HIV status revealed an attenuation of the associations between menopausal status and both low-grade and high-grade cervical neoplasia. In the primary analysis, odds of low-grade and high-grade cervical neoplasia among postmenopausal women were OR = 1.30 (95% CI: 0.84, 2.02) and OR = 1.43 (95% CI: 0.90, 2.28), respectively. In the sensitivity analysis restricted to participants with known HIV status, these attenuated to OR = 1.18 (95% CI: 0.75, 1.85) for low-grade and OR = 1.16 (95% CI: 0.72, 1.86) for high-grade cervical neoplasia. Among HIV-negative women with known status, odds remained elevated but non-significant for both low-grade (OR = 1.42; 95% CI: 0.82, 2.45) and high-grade cervical neoplasia (OR = 1.41; 95% CI: 0.80, 2.46). We present our primary cervical neoplasia results using the imputed data in the tables, with sensitivity analysis results and discussion of their implications reported in the results and discussion sections.

**____________________________________________________________________________________**

**Comment 7:**
Tables 2–4: These tables are not clearly presented. It is suggested to classify HR and LR infections according to the updated criteria from Cervical Cancer Screening, IARC Handbooks of Cancer Prevention, Volume 18 (2022). Analyses should be redone based on this classification: 12 HPV types classified by IARC working group as Group 1 “carcinogenic HPV (cHPV) types” including HPV16, HPV18, HPV45, HPV33, HPV58, HPV31, HPV52, HPV35, HPV59, HPV39, HPV51 and HPV56, where HPV16 had the highest global AF in cervical cancer (61·7%), followed by HPV18 (15.3%), HPV45 (4.8%), HPV33 (3.8%), HPV58 (3.5%), HPV31 (2.8%), and HPV52 (2.8%), between others. In addition, HPV68 in alpha-7 is classified as probably carcinogenic to humans (Group 2A) and last, there are HPV types classified by IARC in Group 2B (possibly carcinogenic), some of which contribute very small attributable fractions and some of which cannot be attributed at all.

**Response:**

Thank-you for this suggestion. As the reviewer is well aware, there are numerous determinations of which HPV types are “high-risk” or “carcinogenic” and which are low-risk/non-carcinogenic, and determination of this status is based upon epidemiologic data and attributable fractions, not biological criteria. IARC (2009) classified 12 types as high-risk for cervical cancer, and in the most recent IARC update (Wei et al, Lancet, 2024), 5 types (HPV 26, 68, 69, 73, and 82) were added and are now considered causal to invasive cervical cancer. In our resubmission, we now categorize HPV 16, 18, 26, 31, 33, 35, 39, 45, 51, 52, 56, 58, 59, 68, 69, 73, and 82 as high risk HPV types, based upon the most recent IARC update (Wei et al, Lancet 2024).

**Comment 8:**
In Table 2, in addition to reporting “any HPV detected,” the authors should specify “any HR-HPV detected,” “any LR-HPV detected,” and multiple infections (HR-HR, HR-LR, LR-LR). HPV types detected in women with precancerous lesions or cancer should be clearly specified, as proportions and distributions vary by group.

**Response:**

Thank you for this suggestion. We have updated Table 2 to include multiple HPV infections and co-infection subcategories (HR & HR-only, LR-only, and HR & LR). For the overall multiple HPV infections outcome, postmenopausal women had significantly higher odds compared to premenopausal women (adjusted OR = 1.94; 95% CI: 1.40, 2.69), with a stronger magnitude of association than for any HPV detection. Among co-infection patterns, HR & LR co-infection was the most common and showed a significant association with menopausal status (adjusted OR = 2.12; 95% CI: 1.48, 3.04). However, the subcategories of HR-only multiple infections (n = 56 total) and LR-only multiple infections (n = 39 total) had insufficient sample sizes to produce stable and/or statistically significant results. Regarding the addition of "any HR-HPV detected" and "any LR-HPV detected" as separate rows in Table 2, we note that these associations are already addressed in Tables 3a and 3b using generalized estimating equations (GEE), which is the more statistically appropriate method for infection-level data given the concurrent testing of multiple HPV types within each participant. We felt that duplicating these results in Table 2 using standard logistic regression could introduce confusion given the methodological differences, and therefore have retained the GEE approach for HR and LR analyses in Tables 3a and 3b. Please note that table numbers have been updated in our new manuscript version.

**Comment 9:**
In Table 3 should be moved to the supplementary material, with HPV detection data presented separately for women with normal results, precancerous lesions, and cancer. When referring to the most common HPV types, this should be contextualized by the presence or absence of precancerous or cancerous lesions.

**Response:**

Women with invasive cancer were not included in this analysis and we have a separate manuscript being revised assessing HPV types in women with invasive cervical cancer. We have previously published a manuscript regarding the prevalence of specific types of HPV in women with and without cervical lesions from one of the cohorts included in the current analysis (see Xi et al, Int J Cancer 2003), as that was a primary aim of the EpiBio study. As assessment of HPV types associated with precancerous lesions is not an aim of the current study, we have chosen to present Table 3 as it was previously presented, not stratified further by those with and without precancerous lesions. However, as suggested, we have moved Table 3 to supplemental material (S1 Table).

**Comment 10:**
In Tables 5 and 6 should be merged into a single table, analyzing associations by comparing women with normal histology results, Low-grade and High-grade lesions, stratified by menopausal status. As in previous tables, HIV-positive and HIV-negative groups should be separated, and all adjustment variables should be included.

**Response:**

As suggested, we have combined Tables 5 and 6 into a single table (Table 4) presenting associations between menopausal status and cervical neoplasia detection, including no neoplasia, any neoplasia, low-grade, and high-grade outcomes, as recommended. Regarding the stratification by HIV status, we note that the association between menopausal status and cervical neoplasia by HIV status is addressed in our statistical analysis through both interaction testing and stratified multivariable logistic regression models. These results are reported in the text of the results section, where we present stratified ORs for HIV-negative and HIV-positive participants separately. We chose not to incorporate this stratification directly into the table, as doing so would substantially increase the table's complexity and reduce readability, particularly given the already small sample sizes within some subgroups. We believe the current presentation provides a clear and complete picture of our findings.

***Discussion Section***

**Comment 11:**
The authors state: “our observed increased detection of high-risk oncogenic HPV among postmenopausal women highlights their vulnerability to future cervical cancer development and reinforces the importance of screening strategies that identify infections early, before cancer can develop.” It is suggested that the authors exercise caution in their conclusions. The kits used for typing are not validated for screening; therefore, detecting a high frequency of HR-HPV does not necessarily imply greater risk of cervical lesion. New control strategies cannot be recommended based on these findings. Instead, longitudinal studies should be conducted to better understand how menopause might influence HR-HPV infection, as well as, frequency and lesion development.

**Response:**

We appreciate your concerns regarding the language we use in our discussion. We strongly disagree with the reviewer’s statement that a high frequency of HR-HPV using our research primers and probes does not necessarily imply greater risk of cervical lesion, as we have published extensively on the use of these research assays and detection of high-grade cervical lesions and invasive cancer (see Xi, Int J Can 2003; Hawes, JID 2003,
Feng, JNCI 2005; Hawes, JNCI 2006). Regardless, we acknowledge that new control strategies cannot be recommended based upon these findings. However, our study does elevate research showing that post-menopausal women are at elevated risk for detection of high-risk oncogenic HPV detection which may place them at elevated risk of pre-invasive cervical lesions and development of invasive cancer, and that cervical cancer screening programs often under-emphasize screening in older women who may be at increased risk of cancer. We agree that longitudinal studies should be conducted to better understand how menopause might influence HR-HPV infection and development of cervical lesions and have updated our discussion accordingly.

**Comment 12:**
Associations with neoplasia are not statistically significant; the authors should clarify whether sample size limited statistical power.

**Response:**

Indeed, we acknowledge that some of our findings were limited by small sample size, especially with regards to the association between menopausal status and prevalent low and high grade cervical lesions. In our revised manuscript, we have added a sentence to our limitations section explicitly noting this, and we are careful throughout the manuscript not to overstate findings that do not reach statistical significance.

**Comment 13:**
External Validity: Finally, the authors are encouraged to enrich the discussion by including comparisons with other African cohorts, which could strengthen external validity, and to explain how their findings align with or diverge from global evidence.

**Response:**

Indeed, our literature search did not identify other African studies which assessed the relationship between menopausal status and detection of cervical HPV and lesions. The only African studies mentioning menopause and cervical disease relate to post-menopausal bleeding as a sign of invasive cervical cancer. As such, we were only able to compare our findings with those from non-African geographies. We have added the need for validation in other African research studies to our discussion section.

**REVIEWER 2**

**Comment 1:**
It would be interesting to understand the effect of time since menopause, since cervical atrophy progresses with time.

**Response:**

In our manuscript we have not assessed time since menopause. Date of last menstrual period was collected in our research studies, but often included only year and/or month and year, and had considerable missing data. It is a good idea to assess time since menopause in future studies and have included this idea in our discussion.

**REVIEWER 3**

**Comment 1:**
The discussion can be modified in relevance to the study results. The wordings such as strong association between HPV infection and menopausal status needs revision. Follow-up details can be added.

**Response:**

We have reviewed the wording in our discussion regarding the associations we observed between HPV infection and menopausal status to confirm that we have not over-stated our results. The term “strong association” is not used in our revised manuscript.
