# Supplementary material for: Impact of menopausal status on human papillomavirus detection and cervical neoplasia in middle-aged Senegalese women
Source: PLoS One. 2026 Sep 11;21(9):e0358080. doi: 10.1371/journal.pone.0358080 (PMC13567829; doi:10.1371/journal.pone.0358080)
Supplement: Supplementary file 3 [file pone.0358080.s005.docx]

Dear Reviewers,

We appreciate the careful consideration you gave to our manuscript for a second review, and the valuable suggestions you shared. Your feedback guided several revisions that have improved the clarity of our results and methods. Below, we respond to each of your comments and indicate how changes were incorporated.

**REVIEWER #4**

**Comment 1:** Handling of missing HIV data: Although the authors conducted sensitivity analyses excluding participants with unknown HIV status, the primary analyses still impute missing HIV status as negative based on the low national prevalence. Given that the missing HIV data are concentrated in the Pikine clinic, where the observed HIV prevalence among tested individuals was 2.7%, this imputation may introduce bias. The authors should present the results of the sensitivity analyses more prominently and clearly state in the abstract and main text that the primary findings for cervical neoplasia were attenuated in these sensitivity analyses.

**Response:** Thank you for your suggestion. The attenuation of cervical neoplasia associations observed in the sensitivity analysis was already addressed in both the results and discussion sections of the manuscript. But to make this more prominent, we have added a sentence to the abstract explicitly noting this attenuation, and have added a statement to the limitations section noting that residual confounding by HIV status cannot be ruled out in our primary cervical neoplasia estimates.

**Comment 2:** Definition of menopausal status: The authors rely primarily on self reported menopausal status and use last menstrual period data only when self report is missing. While this approach is reasonable, the Methods section should more clearly specify the criteria used to define postmenopause. Additionally, the authors should discuss the potential for misclassification due to conditions such as malnutrition or chronic stress that may cause amenorrhea unrelated to menopause.

**Response:** Thank you for your comment. We have added an additional sentence to help clarify how we defined postmenopause in the methods section. Our manuscript already discussed how conditions such as malnutrition or chronic stress could cause loss of menstruation unrelated to menopause, and we have expanded on this by adding statements to our discussion acknowledging that such misclassification could affect the accuracy of our exposure classification and may have impacted the observed associations between menopausal status and HPV and cervical neoplasia detection.

**Comment 3:** Statistical analysis of HPV types: The authors use GEE models for high-risk and low-risk HPV detection, with the reference category as "no HPV detected." This is appropriate. However, the interpretation of the odds ratios for high-risk versus low-risk types should be clarified. Specifically, the GEE model compares the odds of detecting a given HPV type versus not detecting that type. This should be stated explicitly to avoid confusion.

**Response:** Thank you for this suggestion. We have added a clarifying sentence in the statistical analysis section of the methods and have also reworked the opening of the discussion to clarify this when interpreting the type-specific odds ratios.

**Comment 4:** Cervical neoplasia outcomes by HIV status: The stratified analyses by HIV status for cervical neoplasia show divergent results , yet the interaction test was not significant (p=0.14). The authors should emphasize that the study was not powered for stratified analyses. The sensitivity analysis excluding unknown HIV status should also be discussed in more detail, particularly the attenuation of the association.

**Response:** Thank you for this comment. We have added a statement to the discussion explicitly noting that our study was not powered for stratified analysis by HIV status and to interpret with caution. We have also greatly expanded our discussion of the sensitivity analysis and attenuation of cervical neoplasia associations.
