# Supplementary material for: Impact of menopausal status on human papillomavirus detection and cervical neoplasia in middle-aged Senegalese women
Source: PLoS One. 2026 Sep 11;21(9):e0358080. doi: 10.1371/journal.pone.0358080 (PMC13567829; doi:10.1371/journal.pone.0358080)
Supplement: Peer review history

# Peer review: Decision Letter 0

**DOI:** 10.1371/journal.pone.0358080.r001

**Authors:** Ricardo Cobucci

18 May 2026

Dear Dr. Hawes,

Thank you for submitting your manuscript to PLOS ONE. After careful consideration, we feel that it has merit but does not fully meet PLOS ONE’s publication criteria as it currently stands. Therefore, we invite you to submit a revised version of the manuscript that addresses the points raised during the review process.

Dear authors, as you can see, the reviewers have requested substantial revisions to your manuscript. We are certainly willing to reconsider a revised submission, but please know that this is not preliminary acceptance of your paper. When returning your revised manuscript, please be sure to include a point-by-point summary of the suggestions of the reviewers that specifies how and where in the text you have addressed the suggestions.

Please submit your revised manuscript by Jul 02 2026 11:59PM. If you will need more time than this to complete your revisions, please reply to this message or contact the journal office at plosone@plos.org. When you're ready to submit your revision, log on to https://www.editorialmanager.com/pone/ and select the 'Submissions Needing Revision' folder to locate your manuscript file.

- A letter that responds to each point raised by the academic editor and reviewer(s). You should upload this letter as a separate file labeled 'Response to Reviewers'.
- A marked-up copy of your manuscript that highlights changes made to the original version. You should upload this as a separate file labeled 'Revised Manuscript with Track Changes'.
- An unmarked version of your revised paper without tracked changes. You should upload this as a separate file labeled 'Manuscript'.

If you would like to make changes to your financial disclosure, please include your updated statement in your cover letter. Guidelines for resubmitting your figure files are available below the reviewer comments at the end of this letter.

If applicable, we recommend that you deposit your laboratory protocols in protocols.io to enhance the reproducibility of your results. Protocols.io assigns your protocol its own identifier (DOI) so that it can be cited independently in the future. For instructions see: https://journals.plos.org/plosone/s/submission-guidelines#loc-laboratory-protocols. Additionally, PLOS ONE offers an option for publishing peer-reviewed Lab Protocol articles, which describe protocols hosted on protocols.io. Read more information on sharing protocols at https://plos.org/protocols?utm_medium=editorial-email&utm_source=authorletters&utm_campaign=protocols.

As the corresponding author, your ORCID iD is verified in the submission system and will appear in the published article. PLOS supports the use of ORCID, and we encourage all coauthors to register for an ORCID iD and use it as well. Please encourage your coauthors to verify their ORCID iD within the submission system before final acceptance, as unverified ORCID iDs will not appear in the published article. *Only*  the individual author can complete the verification step; PLOS staff *cannot*  verify ORCID iDs on behalf of authors.

We look forward to receiving your revised manuscript.

Kind regards,

Ricardo Ney Oliveira Cobucci, Ph.D

Academic Editor

PLOS One

Journal Requirements:

When submitting your revision, we need you to address these additional requirements.

1. Please ensure that your manuscript meets PLOS ONE's style requirements, including those for file naming. The PLOS ONE style templates can be found at

https://journals.plos.org/plosone/s/file?id=wjVg/PLOSOne_formatting_sample_main_body.pdf and

https://journals.plos.org/plosone/s/file?id=ba62/PLOSOne_formatting_sample_title_authors_affiliations.pdf.

2. Thank you for stating in your Funding Statement:

“Support for the study in which the primary data were collected came from the U.S. National Institutes of Health, National Cancer Institute: grant numbers CA75920, CA97275, and CA111187 to N. B. K. This article reports a secondary data analysis of previously collected data and did not have specific support. “

Please provide an amended statement that declares *all* the funding or sources of support (whether external or internal to your organization) received during this study, as detailed online in our guide for authors at http://journals.plos.org/plosone/s/submit-now.  Please also include the statement “There was no additional external funding received for this study.” in your updated Funding Statement.

Please include your amended Funding Statement within your cover letter. We will change the online submission form on your behalf.

3. Thank you for stating the following financial disclosure:

“Support for the study in which the primary data were collected came from the U.S. National Institutes of Health, National Cancer Institute: grant numbers CA75920, CA97275, and CA111187 to N. B. K. This article reports a secondary data analysis of previously collected data and did not have specific support. “

Please state what role the funders took in the study.  If the funders had no role, please state: "The funders had no role in study design, data collection and analysis, decision to publish, or preparation of the manuscript."

If this statement is not correct you must amend it as needed.

Please include this amended Role of Funder statement in your cover letter; we will change the online submission form on your behalf.

4. Thank you for providing the following Funding Statement:

“S.E.H. reports current grant funding from the Gates Foundation and prior grant funding from the U.S. National Institutes of Health (NIH). R.L.W. reports current grant funding from the U.S. NIH. G. S. G. has received research grants and research support from the US NIH, University of Washington, Gates Foundation, Gilead Sciences, Alere Technologies, Merck & Co, Janssen Pharmaceuticals, Cerus Corporation, ViiV Healthcare, Bristol-Myers Squibb, Roche Molecular Systems, Abbott Molecular Diagnostics, and Theratechnologies/TaiMed Biologics, Inc. All other authors report no conflicts of interest. “

We note that one or more of the authors is affiliated with the funding organization, indicating the funder may have had some role in the design, data collection, analysis or preparation of your manuscript for publication; in other words, the funder played an indirect role through the participation of the co-authors.

If the funding organization did not play a role in the study design, data collection and analysis, decision to publish, or preparation of the manuscript and only provided financial support in the form of authors' salaries and/or research materials, please review your statements relating to the author contributions, and ensure you have specifically and accurately indicated the role(s) that these authors had in your study in the Author Contributions section of the online submission form. Please make any necessary amendments directly within this section of the online submission form.  Please also update your Funding Statement to include the following statement: “The funder provided support in the form of salaries for authors [insert relevant initials], but did not have any additional role in the study design, data collection and analysis, decision to publish, or preparation of the manuscript. The specific roles of these authors are articulated in the ‘author contributions’ section.”

If the funding organization did have an additional role, please state and explain that role within your Funding Statement.

Please also provide an updated Competing Interests Statement declaring this commercial affiliation along with any other relevant declarations relating to employment, consultancy, patents, products in development, or marketed products, etc.

Within your Competing Interests Statement, please confirm that this commercial affiliation does not alter your adherence to all PLOS ONE policies on sharing data and materials by including the following statement: "This does not alter our adherence to  PLOS ONE policies on sharing data and materials.” (as detailed online in our guide for authors http://journals.plos.org/plosone/s/competing-interests). If this adherence statement is not accurate and  there are restrictions on sharing of data and/or materials, please state these. Please note that we cannot proceed with consideration of your article until this information has been declared.

5. When completing the data availability statement of the submission form, you indicated that you will make your data available on acceptance. We strongly recommend all authors decide on a data sharing plan before acceptance, as the process can be lengthy and hold up publication timelines. Please note that, though access restrictions are acceptable now, your entire data will need to be made freely accessible if your manuscript is accepted for publication. This policy applies to all data except where public deposition would breach compliance with the protocol approved by your research ethics board. If you are unable to adhere to our open data policy, please kindly revise your statement to explain your reasoning and we will seek the editor's input on an exemption. Please be assured that, once you have provided your new statement, the assessment of your exemption will not hold up the peer review process.

6. We note that the grant information you provided in the ‘Funding Information’ and ‘Financial Disclosure’ sections do not match.

When you resubmit, please ensure that you provide the correct grant numbers for the awards you received for your study in the ‘Funding Information’ section

7. If the reviewer comments include a recommendation to cite specific previously published works, please review and evaluate these publications to determine whether they are relevant and should be cited. There is no requirement to cite these works unless the editor has indicated otherwise.

[Note: HTML markup is below. Please do not edit.]

Reviewers' comments:

Reviewer's Responses to Questions

**Comments to the Author**

1. Is the manuscript technically sound, and do the data support the conclusions?

Reviewer #1: Partly

Reviewer #2: Yes

Reviewer #3: Yes

**********

2. Has the statistical analysis been performed appropriately and rigorously?

Reviewer #1: No

Reviewer #2: Yes

Reviewer #3: Yes

**********

3. Have the authors made all data underlying the findings in their manuscript fully available?

The PLOS Data policy

Reviewer #1: No

Reviewer #2: Yes

Reviewer #3: Yes

**********

4. Is the manuscript presented in an intelligible fashion and written in standard English?

Reviewer #1: Yes

Reviewer #2: Yes

Reviewer #3: Yes

**********

Reviewer #1: The study, which aimed to analyze the association between postmenopausal status and the presence of HPV infection or cervical lesions, presents major limitations despite its large sample size. The results do not substantiate the stated conclusions, particularly regarding associations with cervical lesions.

Major modifications suggested:

Materials and Methods:

The manuscript states that women from three studies were included. However, it is not clearly specified, for each study, which groups of women were included, how many were excluded, and the reasons for exclusion or inclusion. A flowchart should be provided to visualize these data. It should also be clarified, for each group, how many HIV-positive and HIV-negative women were included. The authors are encouraged to refer to flowchart models such as those presented in:

o Risk factors of human papillomavirus-related cervical lesions in postmenopausal women: a cross-sectional study (BMC Women’s Health, Springer Nature)

o Global prevalence of cervical human papillomavirus in women aged 50 years and older with normal cytology: a systematic review and meta-analysis (The Lancet Microbe).

Inclusion Criteria: The criteria under which women were included in the previous studies should be clarified. Were the same criteria applied, and are the populations comparable? The authors do not specify whether women with precancerous results or those who had received prior treatments were included—data that could bias the results.

Definition of Postmenopause: The definition of postmenopause should be more precise. Although data on the last menstrual period were available, this information was not consistently used and was only considered when not otherwise reported. If such data are available, they should be applied as a criterion to define women in the postmenopausal group.

Secondary Data: HPV and HIV detection, as well as cytology and histopathology results, were secondary data. Therefore, all groups should be detailed in a flowchart within the Materials and Methods section to facilitate comprehension.

Results

In Table 1 should be expanded to include characteristics stratified by menopausal status, incorporating cytology and histology results by group, as well as HIV and HPV detection results. This is essential to visualize the number and proportion of women with these characteristics in each group.

HIV Status Bias: A major bias is that HIV status information is missing for a large group of women. The authors mention that prevalence in Senegal is low (0.4%); however, significant associations with cervical neoplasia detection are observed only in HIV-negative women. Analyses should therefore separate HIV-positive and HIV-negative groups, excluding women with missing HIV data.

Tables 2–4: These tables are not clearly presented. It is suggested to classify HR and LR infections according to the updated criteria from Cervical Cancer Screening, IARC Handbooks of Cancer Prevention, Volume 18 (2022). Analyses should be redone based on this classification: 12 HPV types classified by IARC working group as Group 1 “carcinogenic HPV (cHPV) types” including HPV16, HPV18, HPV45, HPV33, HPV58, HPV31, HPV52, HPV35, HPV59, HPV39, HPV51 and HPV56, where HPV16 had the highest global AF in cervical cancer (61·7%), followed by HPV18 (15.3%), HPV45 (4.8%), HPV33 (3.8%), HPV58 (3.5%), HPV31 (2.8%), and HPV52 (2.8%), between others. In addition, HPV68 in alpha-7 is classified as probably carcinogenic to humans (Group 2A) and last, there are HPV types classified by IARC in Group 2B (possibly carcinogenic), some of which contribute very small attributable fractions and some of which cannot be attributed at all.

In Table 2, in addition to reporting “any HPV detected,” the authors should specify “any HR-HPV detected,” “any LR-HPV detected,” and multiple infections (HR-HR, HR-LR, LR-LR). HPV types detected in women with precancerous lesions or cancer should be clearly specified, as proportions and distributions vary by group.

In Table 3 should be moved to the supplementary material, with HPV detection data presented separately for women with normal results, precancerous lesions, and cancer. When referring to the most common HPV types, this should be contextualized by the presence or absence of precancerous or cancerous lesions.

In Tables 5 and 6 should be merged into a single table, analyzing associations by comparing women with normal histology results, Low-grade and High-grade lesions, stratified by menopausal status. As in previous tables, HIV-positive and HIV-negative groups should be separated, and all adjustment variables should be included.

Discussion

The authors state: “our observed increased detection of high-risk oncogenic HPV among postmenopausal women highlights their vulnerability to future cervical cancer development and reinforces the importance of screening strategies that identify infections early, before cancer can develop.” It is suggested that the authors exercise caution in their conclusions. The kits used for typing are not validated for screening; therefore, detecting a high frequency of HR-HPV does not necessarily imply greater risk of cervical lesion. New control strategies cannot be recommended based on these findings. Instead, longitudinal studies should be conducted to better understand how menopause might influence HR-HPV infection, as well as, frequency and lesion development.

Associations with neoplasia are not statistically significant; the authors should clarify whether sample size limited statistical power.

External Validity: Finally, the authors are encouraged to enrich the discussion by including comparisons with other African cohorts, which could strengthen external validity, and to explain how their findings align with or diverge from global evidence.

Reviewer #2: Elegant work addressing an important knowledge gap. It would be interesting to understand the effect of time since menopause, since cervical atrophy progresses with time. Important implications for public health efforts, particularly in low income settings where risk is high. This is a high impact study with the potential to change clinical practice in settings that have limited screening to premenopausal women.

Reviewer #3: The manuscript is well written. The discussion can be modified in relevance to the study results. The wordings such as strong association between HPV infection and menopausal status needs revision. Follow-up details can be added.

**********

what does this mean?). If published, this will include your full peer review and any attached files.

If you choose “no”, your identity will remain anonymous but your review may still be made public.

**Do you want your identity to be public for this peer review?**  For information about this choice, including consent withdrawal, please see our Privacy Policy

Reviewer #1: No

Reviewer #2: No

Reviewer #3: No

**********

[NOTE: If reviewer comments were submitted as an attachment file, they will be attached to this email and accessible via the submission site. Please log into your account, locate the manuscript record, and check for the action link "View Attachments". If this link does not appear, there are no attachment files.]

To ensure your figures meet our technical requirements, please review our figure guidelines: https://journals.plos.org/plosone/s/figures

You may also use PLOS’s free figure tool, NAAS, to help you prepare publication quality figures: https://journals.plos.org/plosone/s/figures#loc-tools-for-figure-preparation.

NAAS will assess whether your figures meet our technical requirements by comparing each figure against our figure specifications.

# Author comment: Author response to Decision Letter 1

**DOI:** 10.1371/journal.pone.0358080.r002

1 Jul 2026

Dear Reviewers,

We sincerely thank you for your careful evaluation of our manuscript and for their thoughtful and constructive comments. We appreciate the time and effort invested in providing feedback, which has helped us improve the quality and clarity of the manuscript. We have carefully considered each comment and revised the manuscript accordingly. Below, we provide a point-by-point response to each reviewer comment.

________________________________________________________________________

REVIEWER 1

Materials and Methods Section

Comment 1:

The manuscript states that women from three studies were included. However, it is not clearly specified, for each study:

• which groups of women were included

• how many were excluded

• and the reasons for exclusion or inclusion

• It should also be clarified, for each group, how many HIV-positive and HIV-negative women were included.

A flowchart should be provided to visualize these data.

Flow chart examples:

o Risk factors of human papillomavirus-related cervical lesions in postmenopausal women: a cross-sectional study (BMC Women’s Health, Springer Nature)

o Global prevalence of cervical human papillomavirus in women aged 50 years and older with normal cytology: a systematic review and meta-analysis (The Lancet Microbe).

Response:

Thank-you for this suggestion and for providing these two examples. A flow chart (Fig 1) has been added to the submission to illustrate participant selection from each of the three parent studies, including the groups of women included, the numbers and reasons for exclusion, and the numbers of HIV-positive, HIV-negative, and HIV-unknown women included in each of our two primary analyses. A figure title and caption have also been added to the manuscript. Please see the information below.

Below is the Figure which will be included:

Comment 2:

Inclusion Criteria: The criteria under which women were included in the previous studies should be clarified. Were the same criteria applied, and are the populations comparable? The authors do not specify whether women with precancerous results or those who had received prior treatments were included—data that could bias the results.

(similar to the ‘and the reasons for exclusion or inclusion above)

Response:

Inclusion criteria for the three parent studies were as follows: The “Epidemiology and Biology of Cervical Neoplasia” study enrolled women aged 35 and older from two study clinics: 1 - a primary care clinic (Centre de Santé Dominique de Pikine) and 2 - an oncology clinic (Hôpital Aristide Le Dantec, Institute Curie, Dakar). The “New Approaches to Cervical Cancer Control” study enrolled women aged 18 and older from two study clinics: 1 - an infectious disease clinic (CHNU-Fann, SMIT, Dakar) and 2 - 2 - an oncology clinic (Hôpital Aristide Le Dantec, Institute Curie, Dakar). Finally, the “DNA Hypermethylation in Cervical Cancer” study enrolled women 18 and older from two study clinics: 1 - a primary care clinic (Centre de Santé Dominique de Pikine) and 2 - an infectious disease clinic (CHNU-Fann, SMIT, Dakar). There were no further inclusion or exclusion criteria for entry into the parent studies. Women enrolling at the Dantec oncology clinic were referred to that clinic with presumed cancer (and if they had cancer, were excluded from the present secondary data analysis). Women enrolling at Pikine or Fann presented for various other reasons not related to cervical cancer and outside of our study, cervical cancer screening was not offered at those clinics. As cervical cancer screening is rare in Senegal, it is possible that some women were previous screened and/or treated for prior cervical lesions, but none presented for follow-up of cervical screening and/or treatment at the time of enrollment into our studies. We have added additional information to reflect the study eligibility criteria for the three parent studies to the methods section.

Comment 3:

Definition of Postmenopause: The definition of postmenopause should be more precise. Although data on the last menstrual period were available, this information was not consistently used and was only considered when not otherwise reported. If such data are available, they should be applied as a criterion to define women in the postmenopausal group.

Response:

Thank-you for this suggestion. As noted in the methods section, Information on whether a women was postmenopausal or not was obtained from self-report during the baseline study interview and was used as the primary determination of menopausal status. Information regarding last menstrual period (LMP) was also obtained during the interview and was used only when self-reported menopausal status was answered as “don’t know” or missing as LMP was also self-reported, seemed less reliable (often only month or year was known), and was often missing. If a woman was enrolled into follow-up, missing menopausal status was obtained from a future visit (n=52). Participants were excluded if menopausal status could not be obtained from these three sources (n=44). Information regarding the number of women excluded for these reasons combined has been included in Figure 1.

Comment 4:

Secondary Data: HPV and HIV detection, as well as cytology and histopathology results, were secondary data. Therefore, all groups should be detailed in a flow chart within the Materials and Methods section to facilitate comprehension.

Response:

Cervical samples were obtained for cytology and HPV detection in all study protocols. However, as some samples were inadequate for cytologic evaluation and/or HPV DNA testing, participants were excluded from analyses. Information on these exclusions and those with missing HIV testing is now included in the flow chart of study participants (new Figure 1).

Results Section

Comment 5:

Table 1 should be expanded to include characteristics stratified by menopausal status, incorporating cytology and histology results by group, as well as HIV and HPV detection results. This is essential to visualize the number and proportion of women with these characteristics in each group.

Response:

We appreciate this suggestion by the reviewer. HIV status was already in Table 1 and remains reported there in our revision. However, as HPV and cervical abnormalities are the outcomes of interest, we have reserved reporting of those outcomes as before, in later tables. HPV overall (Table 2) and by specific type and grouped by low- and high-risk types (Table 3) are clearly presented, stratified by menopausal status. We feel that adding all of this information to Table 1 would be unwieldy and would result in a Table 1 beyond what is typical, covering multiple pages. Similarly, in Table 4, we present the combined cytology/histology results, again stratified by menopausal status. Please note that Tables have been renumbered in our revised manuscript.

Comment 6:

HIV Status Bias: A major bias is that HIV status information is missing for a large group of women. The authors mention that prevalence in Senegal is low (0.4%); however, significant associations with cervical neoplasia detection are observed only in HIV-negative women. Analyses should therefore separate HIV-positive and HIV-negative groups, excluding women with missing HIV data.

Response:

Thank you for sharing your concerns about our approach to imputing HIV status as negative in the subset of subjects on whom HIV testing was not conducted. HIV was considered a confounder in analyses related to HPV but was found to be a potential effect modifier in the association between menopausal status and cervical neoplasia. To address this concern, we conducted sensitivity analyses excluding participants with unknown HIV status for both our HPV and cervical neoplasia analyses, and have added the results of these sensitivity analyses to the results section. With regards to HPV detection, the sensitivity analysis results were consistent with our primary findings. The OR for any HPV detection associated with menopausal status changed minimally from 1.48 to 1.51 when participants with unknown HIV status were excluded, and results for both high-risk and low-risk HPV types were similarly unchanged. We therefore continue to present the primary HPV analyses using the imputed data in the tables, with sensitivity analysis results reported in the text. With regards to cervical neoplasia, the sensitivity analysis excluding participants with unknown HIV status revealed an attenuation of the associations between menopausal status and both low-grade and high-grade cervical neoplasia. In the primary analysis, odds of low-grade and high-grade cervical neoplasia among postmenopausal women were OR = 1.30 (95% CI: 0.84, 2.02) and OR = 1.43 (95% CI: 0.90, 2.28), respectively. In the sensitivity analysis restricted to participants with known HIV status, these attenuated to OR = 1.18 (95% CI: 0.75, 1.85) for low-grade and OR = 1.16 (95% CI: 0.72, 1.86) for high-grade cervical neoplasia. Among HIV-negative women with known status, odds remained elevated but non-significant for both low-grade (OR = 1.42; 95% CI: 0.82, 2.45) and high-grade cervical neoplasia (OR = 1.41; 95% CI: 0.80, 2.46). We present our primary cervical neoplasia results using the imputed data in the tables, with sensitivity analysis results and discussion of their implications reported in the results and discussion sections.

____________________________________________________________________________________

Comment 7:

Tables 2–4: These tables are not clearly presented. It is suggested to classify HR and LR infections according to the updated criteria from Cervical Cancer Screening, IARC Handbooks of Cancer Prevention, Volume 18 (2022). Analyses should be redone based on this classification: 12 HPV types classified by IARC working group as Group 1 “carcinogenic HPV (cHPV) types” including HPV16, HPV18, HPV45, HPV33, HPV58, HPV31, HPV52, HPV35, HPV59, HPV39, HPV51 and HPV56, where HPV16 had the highest global AF in cervical cancer (61·7%), followed by HPV18 (15.3%), HPV45 (4.8%), HPV33 (3.8%), HPV58 (3.5%), HPV31 (2.8%), and HPV52 (2.8%), between others. In addition, HPV68 in alpha-7 is classified as probably carcinogenic to humans (Group 2A) and last, there are HPV types classified by IARC in Group 2B (possibly carcinogenic), some of which contribute very small attributable fractions and some of which cannot be attributed at all.

Response:

Thank-you for this suggestion. As the reviewer is well aware, there are numerous determinations of which HPV types are “high-risk” or “carcinogenic” and which are low-risk/non-carcinogenic, and determination of this status is based upon epidemiologic data and attributable fractions, not biological criteria. IARC (2009) classified 12 types as high-risk for cervical cancer, and in the most recent IARC update (Wei et al, Lancet, 2024), 5 types (HPV 26, 68, 69, 73, and 82) were added and are now considered causal to invasive cervical cancer. In our resubmission, we now categorize HPV 16, 18, 26, 31, 33, 35, 39, 45, 51, 52, 56, 58, 59, 68, 69, 73, and 82 as high risk HPV types, based upon the most recent IARC update (Wei et al, Lancet 2024).

Comment 8:

In Table 2, in addition to reporting “any HPV detected,” the authors should specify “any HR-HPV detected,” “any LR-HPV detected,” and multiple infections (HR-HR, HR-LR, LR-LR). HPV types detected in women with precancerous lesions or cancer should be clearly specified, as proportions and distributions vary by group.

Response:

Thank you for this suggestion. We have updated Table 2 to include multiple HPV infections and co-infection subcategories (HR & HR-only, LR-only, and HR & LR). For the overall multiple HPV infections outcome, postmenopausal women had significantly higher odds compared to premenopausal women (adjusted OR = 1.94; 95% CI: 1.40, 2.69), with a stronger magnitude of association than for any HPV detection. Among co-infection patterns, HR & LR co-infection was the most common and showed a significant association with menopausal status (adjusted OR = 2.12; 95% CI: 1.48, 3.04). However, the subcategories of HR-only multiple infections (n = 56 total) and LR-only multiple infections (n = 39 total) had insufficient sample sizes to produce stable and/or statistically significant results. Regarding the addition of "any HR-HPV detected" and "any LR-HPV detected" as separate rows in Table 2, we note that these associations are already addressed in Tables 3a and 3b using generalized estimating equations (GEE), which is the more statistically appropriate method for infection-level data given the concurrent testing of multiple HPV types within each participant. We felt that duplicating these results in Table 2 using standard logistic regression could introduce confusion given the methodological differences, and therefore have retained the GEE approach for HR and LR analyses in Tables 3a and 3b. Please note that table numbers have been updated in our new manuscript version.

Comment 9:

In Table 3 should be moved to the supplementary material, with HPV detection data presented separately for women with normal results, precancerous lesions, and cancer. When referring to the most common HPV types, this should be contextualized by the presence or absence of precancerous or cancerous lesions.

Response:

Women with invasive cancer were not included in this analysis and we have a separate manuscript being revised assessing HPV types in women with invasive cervical cancer. We have previously published a manuscript regarding the prevalence of specific types of HPV in women with and without cervical lesions from one of the cohorts included in the current analysis (see Xi et al, Int J Cancer 2003), as that was a primary aim of the EpiBio study. As assessment of HPV types associated with precancerous lesions is not an aim of the current study, we have chosen to present Table 3 as it was previously presented, not stratified further by those with and without precancerous lesions. However, as suggested, we have moved Table 3 to supplemental material (S1 Table).

Comment 10:

In Tables 5 and 6 should be merged into a single table, analyzing associations by comparing women with normal histology results, Low-grade and High-grade lesions, stratified by menopausal status. As in previous tables, HIV-positive and HIV-negative groups should be separated, and all adjustment variables should be included.

Response:

As suggested, we have combined Tables 5 and 6 into a single table (Table 4) presenting associations between menopausal status and cervical neoplasia detection, including no neoplasia, any neoplasia, low-grade, and high-grade outcomes, as recommended. Regarding the stratification by HIV status, we note that the association between menopausal status and cervical neoplasia by HIV status is addressed in our statistical analysis through both interaction testing and stratified multivariable logistic regression models. These results are reported in the text of the results section, where we present stratified ORs for HIV-negative and HIV-positive participants separately. We chose not to incorporate this stratification directly into the table, as doing so would substantially increase the table's complexity and reduce readability, particularly given the already small sample sizes within some subgroups. We believe the current presentation provides a clear and complete picture of our findings.

Discussion Section

Comment 11:

The authors state: “our observed increased detection of high-risk oncogenic HPV among postmenopausal women highlights their vulnerability to future cervical cancer development and reinforces the importance of screening strategies that identify infections early, before cancer can develop.” It is suggested that the authors exercise caution in their conclusions. The kits used for typing are not validated for screening; therefore, detecting a high frequency of HR-HPV does not necessarily imply greater risk of cervical lesion. New control strategies cannot be recommended based on these findings. Instead, longitudinal studies should be conducted to better understand how menopause might influence HR-HPV infection, as well as, frequency and lesion development.

Response:

We appreciate your co

Attachment Submitted filename: Response to Reviewers_June 30.docx

# Peer review: Decision Letter 1

**DOI:** 10.1371/journal.pone.0358080.r003

**Authors:** Ricardo Cobucci

4 Aug 2026

Dear Dr. Hawes,

Thank you for submitting your manuscript to PLOS ONE. After careful consideration, we feel that it has merit but does not fully meet PLOS ONE’s publication criteria as it currently stands. Therefore, we invite you to submit a revised version of the manuscript that addresses the points raised during the review process.

Please submit your revised manuscript by Sep 18 2026 11:59PM. If you will need more time than this to complete your revisions, please reply to this message or contact the journal office at plosone@plos.org. When you're ready to submit your revision, log on to https://www.editorialmanager.com/pone/ and select the 'Submissions Needing Revision' folder to locate your manuscript file.

- A letter that responds to each point raised by the academic editor and reviewer(s). You should upload this letter as a separate file labeled 'Response to Reviewers'.
- A marked-up copy of your manuscript that highlights changes made to the original version. You should upload this as a separate file labeled 'Revised Manuscript with Track Changes'.
- An unmarked version of your revised paper without tracked changes. You should upload this as a separate file labeled 'Manuscript'.

If you would like to make changes to your financial disclosure, please include your updated statement in your cover letter. Guidelines for resubmitting your figure files are available below the reviewer comments at the end of this letter.

If applicable, we recommend that you deposit your laboratory protocols in protocols.io to enhance the reproducibility of your results. Protocols.io assigns your protocol its own identifier (DOI) so that it can be cited independently in the future. For instructions see: https://journals.plos.org/plosone/s/submission-guidelines#loc-laboratory-protocols. Additionally, PLOS ONE offers an option for publishing peer-reviewed Lab Protocol articles, which describe protocols hosted on protocols.io. Read more information on sharing protocols at https://plos.org/protocols?utm_medium=editorial-email&utm_source=authorletters&utm_campaign=protocols.

As the corresponding author, your ORCID iD is verified in the submission system and will appear in the published article. PLOS supports the use of ORCID, and we encourage all coauthors to register for an ORCID iD and use it as well. Please encourage your coauthors to verify their ORCID iD within the submission system before final acceptance, as unverified ORCID iDs will not appear in the published article. *Only*  the individual author can complete the verification step; PLOS staff *cannot*  verify ORCID iDs on behalf of authors.

We look forward to receiving your revised manuscript.

Kind regards,

Ricardo Ney Oliveira Cobucci, Ph.D

Academic Editor

PLOS One

Journal Requirements:

If the reviewer comments include a recommendation to cite specific previously published works, please review and evaluate these publications to determine whether they are relevant and should be cited. There is no requirement to cite these works unless the editor has indicated otherwise.

Please review your reference list to ensure that it is complete and correct. If you have cited papers that have been retracted, please include the rationale for doing so in the manuscript text, or remove these references and replace them with relevant current references. Any changes to the reference list should be mentioned in the rebuttal letter that accompanies your revised manuscript. If you need to cite a retracted article, indicate the article’s retracted status in the References list and also include a citation and full reference for the retraction notice.

[Note: HTML markup is below. Please do not edit.]

Reviewers' comments:

Reviewer's Responses to Questions

**Comments to the Author**

Reviewer #3: All comments have been addressed

Reviewer #4: All comments have been addressed

**********

2. Is the manuscript technically sound, and do the data support the conclusions?

Reviewer #3: Yes

Reviewer #4: Yes

**********

3. Has the statistical analysis been performed appropriately and rigorously?

Reviewer #3: Yes

Reviewer #4: Yes

**********

4. Have the authors made all data underlying the findings in their manuscript fully available?

The PLOS Data policy

Reviewer #3: Yes

Reviewer #4: No

**********

5. Is the manuscript presented in an intelligible fashion and written in standard English?

Reviewer #3: Yes

Reviewer #4: Yes

**********

Reviewer #3: The recommended suggestions in the first review has been incorporated in the manuscript and the necessary changes have been made by the author.

Reviewer #4: The authors have provided a thorough and generally satisfactory response to the previous round of review. The addition of a participant flow diagram , clarification of inclusion criteria, updates to HPV type classification according to the latest IARC criteria, and sensitivity analyses excluding participants with unknown HIV status have significantly improved the manuscript.The study addresses an important and under-researched topic,HPV and cervical neoplasia among postmenopausal women in West Africa,its findings carry meaningful public health implications for cervical cancer screening in low-resource settings.However, several issues remain that should be addressed before the manuscript can be accepted for publication.

1.Handling of missing HIV data:Although the authors conducted sensitivity analyses excluding participants with unknown HIV status, the primary analyses still impute missing HIV status as negative based on the low national prevalence . Given that the missing HIV data are concentrated in the Pikine clinic, where the observed HIV prevalence among tested individuals was 2.7%, this imputation may introduce bias. The authors should present the results of the sensitivity analyses more prominently and clearly state in the abstract and main text that the primary findings for cervical neoplasia were attenuated in these sensitivity analyses.

2.Definition of menopausal status:The authors rely primarily on self reported menopausal status and use last menstrual period data only when self report is missing. While this approach is reasonable, the Methods section should more clearly specify the criteria used to define postmenopause . Additionally, the authors should discuss the potential for misclassification due to conditions such as malnutrition or chronic stress that may cause amenorrhea unrelated to menopause.

3.Statistical analysis of HPV types:The authors use GEE models for high-risk and low-risk HPV detection, with the reference category as "no HPV detected." This is appropriate. However, the interpretation of the odds ratios for high-risk versus low-risk types should be clarified. Specifically, the GEE model compares the odds of detecting a given HPV type versus not detecting that type. This should be stated explicitly to avoid confusion.

4.Cervical neoplasia outcomes by HIV status:The stratified analyses by HIV status for cervical neoplasia show divergent results , yet the interaction test was not significant (p=0.14). The authors should emphasize that the study was not powered for stratified analyses. The sensitivity analysis excluding unknown HIV status should also be discussed in more detail, particularly the attenuation of the association.

**********

what does this mean?). If published, this will include your full peer review and any attached files.

If you choose “no”, your identity will remain anonymous but your review may still be made public.

**Do you want your identity to be public for this peer review?**  For information about this choice, including consent withdrawal, please see our Privacy Policy

Reviewer #3: No

Reviewer #4: No

**********

[NOTE: If reviewer comments were submitted as an attachment file, they will be attached to this email and accessible via the submission site. Please log into your account, locate the manuscript record, and check for the action link "View Attachments". If this link does not appear, there are no attachment files.]

To ensure your figures meet our technical requirements, please review our figure guidelines: https://journals.plos.org/plosone/s/figures

You may also use PLOS’s free figure tool, NAAS, to help you prepare publication quality figures: https://journals.plos.org/plosone/s/figures#loc-tools-for-figure-preparation.

NAAS will assess whether your figures meet our technical requirements by comparing each figure against our figure specifications.

# Author comment: Author response to Decision Letter 2

**DOI:** 10.1371/journal.pone.0358080.r004

25 Aug 2026

Dear Reviewers,

We appreciate the careful consideration you gave to our manuscript for a second review, and the valuable suggestions you shared. Your feedback guided several revisions that have improved the clarity of our results and methods. Below, we respond to each of your comments and indicate how changes were incorporated.

REVIEWER #4

Comment 1: Handling of missing HIV data: Although the authors conducted sensitivity analyses excluding participants with unknown HIV status, the primary analyses still impute missing HIV status as negative based on the low national prevalence. Given that the missing HIV data are concentrated in the Pikine clinic, where the observed HIV prevalence among tested individuals was 2.7%, this imputation may introduce bias. The authors should present the results of the sensitivity analyses more prominently and clearly state in the abstract and main text that the primary findings for cervical neoplasia were attenuated in these sensitivity analyses.

Response: Thank you for your suggestion. The attenuation of cervical neoplasia associations observed in the sensitivity analysis was already addressed in both the results and discussion sections of the manuscript. But to make this more prominent, we have added a sentence to the abstract explicitly noting this attenuation, and have added a statement to the limitations section noting that residual confounding by HIV status cannot be ruled out in our primary cervical neoplasia estimates.

Comment 2: Definition of menopausal status: The authors rely primarily on self reported menopausal status and use last menstrual period data only when self report is missing. While this approach is reasonable, the Methods section should more clearly specify the criteria used to define postmenopause. Additionally, the authors should discuss the potential for misclassification due to conditions such as malnutrition or chronic stress that may cause amenorrhea unrelated to menopause.

Response: Thank you for your comment. We have added an additional sentence to help clarify how we defined postmenopause in the methods section. Our manuscript already discussed how conditions such as malnutrition or chronic stress could cause loss of menstruation unrelated to menopause, and we have expanded on this by adding statements to our discussion acknowledging that such misclassification could affect the accuracy of our exposure classification and may have impacted the observed associations between menopausal status and HPV and cervical neoplasia detection.

Comment 3: Statistical analysis of HPV types: The authors use GEE models for high-risk and low-risk HPV detection, with the reference category as "no HPV detected." This is appropriate. However, the interpretation of the odds ratios for high-risk versus low-risk types should be clarified. Specifically, the GEE model compares the odds of detecting a given HPV type versus not detecting that type. This should be stated explicitly to avoid confusion.

Response: Thank you for this suggestion. We have added a clarifying sentence in the statistical analysis section of the methods and have also reworked the opening of the discussion to clarify this when interpreting the type-specific odds ratios.

Comment 4: Cervical neoplasia outcomes by HIV status: The stratified analyses by HIV status for cervical neoplasia show divergent results , yet the interaction test was not significant (p=0.14). The authors should emphasize that the study was not powered for stratified analyses. The sensitivity analysis excluding unknown HIV status should also be discussed in more detail, particularly the attenuation of the association.

Response: Thank you for this comment. We have added a statement to the discussion explicitly noting that our study was not powered for stratified analysis by HIV status and to interpret with caution. We have also greatly expanded our discussion of the sensitivity analysis and attenuation of cervical neoplasia associations.

Attachment Submitted filename: Response to Reviewers_August 21 2026.docx

# Editor report: Decision Letter 2

**DOI:** 10.1371/journal.pone.0358080.r005

**Authors:** Ricardo Cobucci

26 Aug 2026

Impact of menopausal status on human papillomavirus detection and cervical neoplasia in middle-aged Senegalese women

PONE-D-25-63102R2

Dear Dr. Hawes,

We’re pleased to inform you that your manuscript has been judged scientifically suitable for publication and will be formally accepted for publication once it meets all outstanding technical requirements.

Within one week, you’ll receive an e-mail detailing the required amendments. When these have been addressed, you’ll receive a formal acceptance letter and your manuscript will be scheduled for publication.

An invoice will be generated when your article is formally accepted. Please note, if your institution has a publishing partnership with PLOS and your article meets the relevant criteria, all or part of your publication costs will be covered. Please make sure your user information is up-to-date by logging into Editorial Manager at Editorial Manager® and clicking the ‘Update My Information' link at the top of the page. For questions related to billing, please contact billing support.

If your institution or institutions have a press office, please notify them about your upcoming paper to help maximize its impact. If they’ll be preparing press materials, please inform our press team as soon as possible -- no later than 48 hours after receiving the formal acceptance. Your manuscript will remain under strict press embargo until 2 pm Eastern Time on the date of publication. For more information, please contact onepress@plos.org.

Kind regards,

Ricardo Ney Oliveira Cobucci, Ph.D

Academic Editor

PLOS One

Additional Editor Comments (optional):

The authors followed the reviewers' recommendations, and the manuscript is ready for publication.

Reviewers' comments:

# Editor report: Acceptance letter

**DOI:** 10.1371/journal.pone.0358080.r006

**Authors:** Ricardo Cobucci

PONE-D-25-63102R2

PLOS One

Dear Dr. Hawes,

I'm pleased to inform you that your manuscript has been deemed suitable for publication in PLOS One. Congratulations! Your manuscript is now being handed over to our production team.

At this stage, our production department will prepare your paper for publication. This includes ensuring the following:

* All references, tables, and figures are properly cited

* All relevant supporting information is included in the manuscript submission,

* There are no issues that prevent the paper from being properly typeset

You will receive further instructions from the production team, including instructions on how to review your proof when it is ready. Please keep in mind that we are working through a large volume of accepted articles, so please give us a few days to review your paper and let you know the next and final steps.

Lastly, if your institution or institutions have a press office, please let them know about your upcoming paper now to help maximize its impact. If they'll be preparing press materials, please inform our press team within the next 48 hours. Your manuscript will remain under strict press embargo until 2 pm Eastern Time on the date of publication. For more information, please contact onepress@plos.org.

You will receive an invoice from PLOS for your publication fee after your manuscript has reached the completed accept phase. If you receive an email requesting payment before acceptance or for any other service, this may be a phishing scheme. Learn how to identify phishing emails and protect your accounts at https://explore.plos.org/phishing.

If we can help with anything else, please email us at customercare@plos.org.

Thank you for submitting your work to PLOS One and supporting open access.

Kind regards,

PLOS One Editorial Office Staff

on behalf of

PROFESSOR Ricardo Ney Oliveira Cobucci

Academic Editor

PLOS One
